# HiPS-Endothelial Cells Acquire Cardiac Endothelial Phenotype in Co-culture With hiPS-Cardiomyocytes

**DOI:** 10.3389/fcell.2021.715093

**Published:** 2021-08-06

**Authors:** Emmi Helle, Minna Ampuja, Alexandra Dainis, Laura Antola, Elina Temmes, Erik Tolvanen, Eero Mervaala, Riikka Kivelä

**Affiliations:** ^1^Stem Cells and Metabolism Research Program, Faculty of Medicine, University of Helsinki, Helsinki, Finland; ^2^New Children’s Hospital, Pediatric Research Center, Helsinki University Hospital, Helsinki, Finland; ^3^Department of Genetics, Stanford University, Stanford, CA, United States; ^4^Department of Pharmacology, Faculty of Medicine, University of Helsinki, Helsinki, Finland; ^5^Samaria Health Centre, Espoo, Finland; ^6^Wihuri Research Institute, Helsinki, Finland

**Keywords:** human induced pluripotent stem cells, cardiomyocytes, endothelial cells, co-culture, single cell RNA sequencing

## Abstract

Cell-cell interactions are crucial for organ development and function. In the heart, endothelial cells engage in bidirectional communication with cardiomyocytes regulating cardiac development and growth. We aimed to elucidate the organotypic development of cardiac endothelial cells and cardiomyocyte and endothelial cell crosstalk using human induced pluripotent stem cells (hiPSC). Single-cell RNA sequencing was performed with hiPSC-derived cardiomyocytes (hiPS-CMs) and endothelial cells (hiPS-ECs) in mono- and co-culture. The presence of hiPS-CMs led to increased expression of transcripts related to vascular development and maturation, cardiac development, as well as cardiac endothelial cell and endocardium-specific genes in hiPS-ECs. Interestingly, co-culture induced the expression of cardiomyocyte myofibrillar genes and MYL7 and MYL4 protein expression was detected in hiPS-ECs. Major regulators of BMP- and Notch-signaling pathways were induced in both cell types in co-culture. These results reflect the findings from animal studies and extend them to human endothelial cells, demonstrating the importance of EC-CM interactions during development.

## Introduction

Endothelial cells (ECs) line the interior surfaces of blood vessels throughout the whole body. ECs are metabolically active, control blood flow and vasomotor tone, and regulate the transport of nutrients and waste. *In vivo*, endothelial cells acquire organotypic identities according to diverse stimuli from blood flow, hormones, and crosstalk signals from the parenchymal cells (le Noble et al., 2004; Red-Horse et al., 2007; Atkins et al., 2011). Arterial, venous, and lymphatic ECs exhibit their specific gene expression profiles, as do ECs in different organs in the body. The organotypic heterogeneity of endothelial cells is now considered as their core property, but the mechanisms regulating the organotypic features are still largely unknown. Primary ECs, however, have been demonstrated to lose their tissue identity within a couple of days in *in vitro* culture after isolation, highlighting the importance of the niche signaling (Nolan et al., 2013; Coppiello et al., 2015).

Endothelial cells comprise a significant proportion of the cells in the heart (Pinto et al., 2016). Endothelial cell-cardiomyocyte (CM) crosstalk is an important process during cardiac development, but also in adult life (Talman and Kivelä, 2018). Several animal studies have now identified organ-specific genes/proteins in ECs isolated from various mouse organs (Nolan et al., 2013; Coppiello et al., 2015; Jambusaria et al., 2020; Paik et al., 2020), including cardiac EC-specific genes. Interestingly, recent reports have indicated that ECs can acquire expression of genes typical for the parenchymal cells, such as cardiomyocyte myofibril genes in the heart endothelial cells and synaptic vesicle genes in the brain endothelium (Jambusaria et al., 2020; Yucel et al., 2020). However, it is not currently known if this applies to human cells.

Since the method of deriving human induced pluripotent stem cells (hiPSCs) from adult somatic cells was developed (Takahashi and Yamanaka, 2006), numerous different cell types have been differentiated from these cells, including ECs and CMs. HiPSCs are a powerful tool to study specific diseases in patient-derived cells (Hamazaki et al., 2017). However, hiPS-EC differentiation protocols often result in a cell population expressing immature EC markers, potentially resulting in plasticity to further develop to, or even transfer between arterial and venous phenotypes (Rufaihah et al., 2013; Ikuno et al., 2017). Similarly, most hiPS-CM differentiation protocols result in an immature cell population with an embryonic-like gene expression pattern (Yang et al., 2014). Recently, co-culture and cardiac 3D microtissue models have demonstrated that the presence of fibroblasts and ECs led to more mature hiPSC-derived cardiomyocytes (hiPS-CMs) with improved sarcomeric structures, and enhanced contractility (Matsuda et al., 2018; Dunn et al., 2019; Giacomelli et al., 2020). However, the effect of CMs to the EC phenotype in co-culture is less studied.

Here, we set out to examine the interaction between hiPS-ECs and hiPS-CMs in a co-culture system to model organotypic vasculature development in the human heart. Our hypothesis was that co-culture remodels hiPS-EC and hiPS-CM transcriptomes and results in an *in vitro* model that is more relevant for cardiac disease modeling than either cell type alone. We demonstrate that already after 48 h in co-culture, ECs acquire features of cardiac-specific ECs and increase the expression of genes related to cardiovascular development and myofibrillar contraction. Co-culture also improved the maturity and homogeneity of the hiPS-ECs. Our results indicate that co-culture of hiPS-ECs and hiPS-CMs provides physiological cell culture conditions for hiPS-ECs, resembling the activation of the same signaling pathways as demonstrated in *in vivo* animal models.

## Results

### Characterization of the hiPS-CMs and hiPS-ECs

**FIGURE 1:**
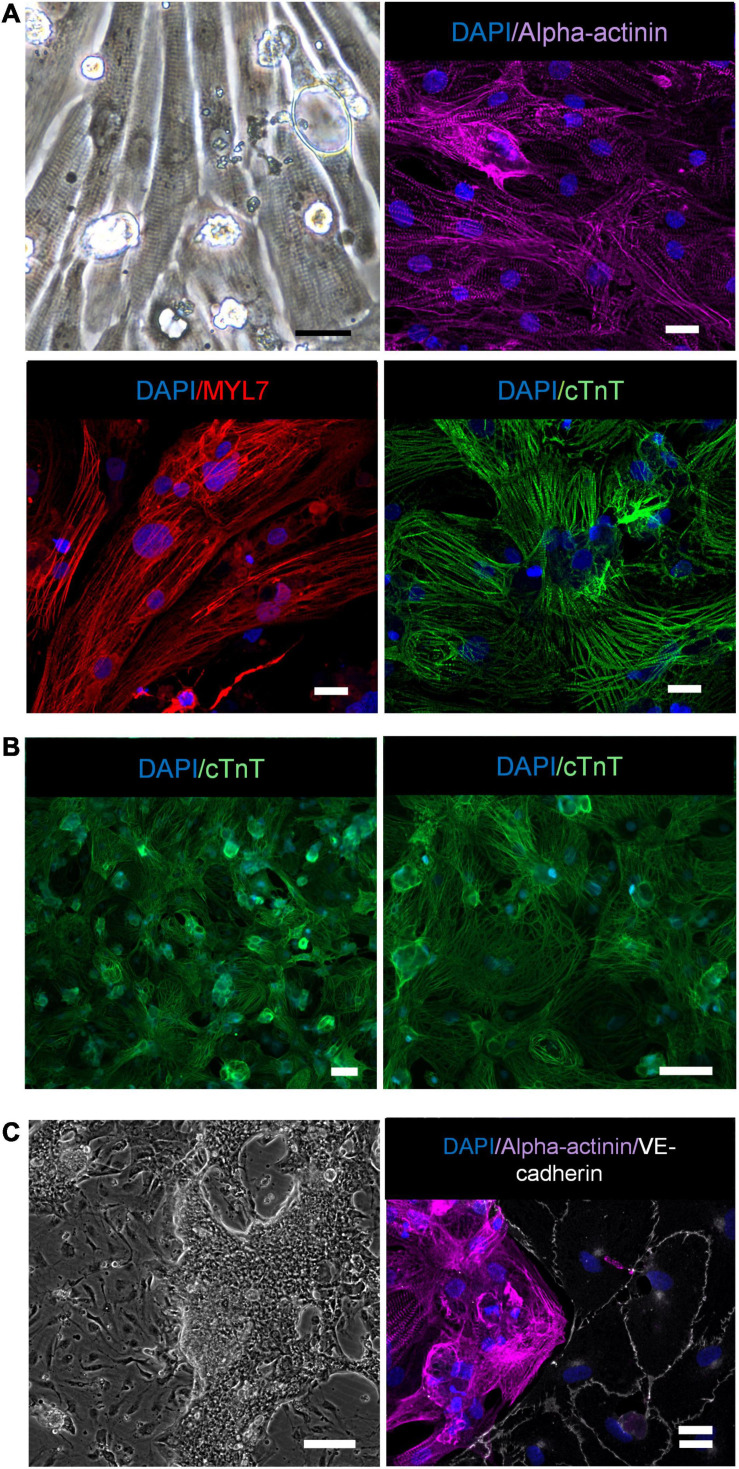
CM characterization. **(A)** Light-microscope image of hiPS-CMs (scale bar 25 μm), immunofluorescence staining of hiPS-CMs with alpha-actinin, MYL7 and cTnT (scale bar 20 μm). **(B)** Images at lower magnification illustrate the purity of the CM population (scale bar 50 μm). **(C)** Light microscope image of hiPS-EC and hiPS-CM co-culture (scale bar 200 μm), immunofluorescence staining hiPS-EC and hiPS-CM co-culture with alpha-actinin and VE-cadherin (scale bar 20 μm).

We have recently reported that the hiPS-ECs produced by the protocol used in the present study have a cobblestone morphology, they strongly express VE-cadherin and PECAM-1 in cell-cell junctions, they take up oxidized LDL, are capable of forming tubes in Matrigel and are highly responsive to flow (Helle et al., 2020). Here, light microscopy and alpha-actinin (ACTN2), myosin light chain 7 (MYL7), and cardiac troponin (cTnT) immunofluorescence stainings of the hiPS-CMs show clear sarcomeric structures (Figure 1A), and images at lower magnification illustrate the purity of the CM population (Figure 1B). The hiPS-ECs demonstrated high levels of VE-cadherin in EC junctions in co-cultures (Figure 1C). The regular sarcomeric structures were also seen in the beating cardiomyocytes (Supplementary Movie I). The beating cardiomyocytes formed dense cell clusters between the ECs, and the clusters were connected to each other by CMs bridging over the EC layer (Figure 1C and Supplementary Movie II). The qPCR analysis showed consistent increase in the expression of cardiac genes in the hiPS-CMs during differentiation in both studied hiPS cell lines (Supplementary Figure 1).

**FIGURE 2:**
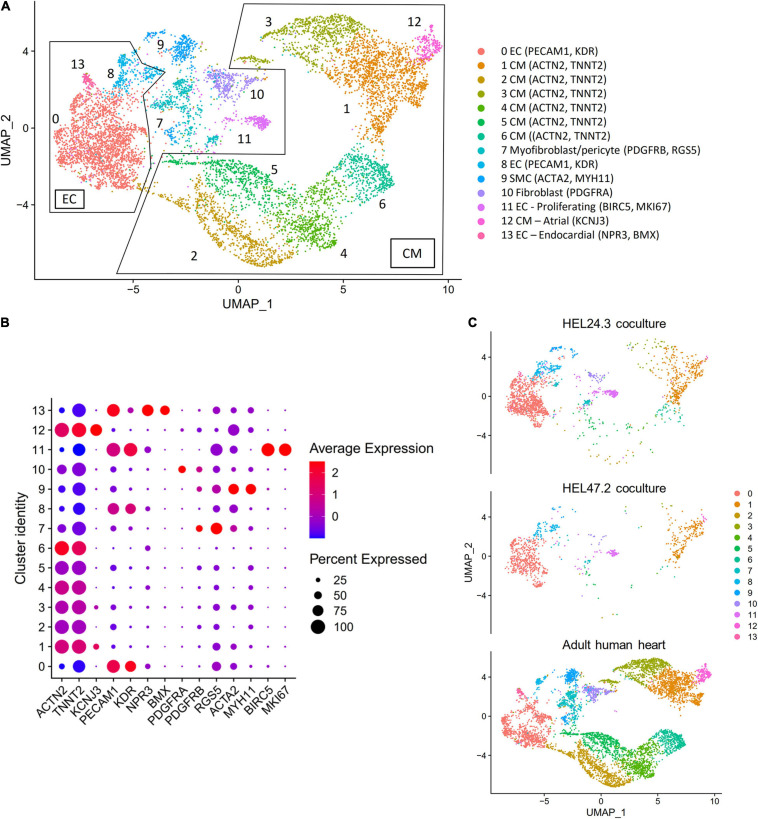
Co-culture hiPS-CM/EC transcriptomics compared to the human heart, with single cell transcriptome data of three combined samples [HEL24.3 co-culture of hiPS-CMs and hiPS-ECs, HEL47.2 co-culture of hiPS-CMs and hiPS-ECs, and human heart derived cells from a previously published study (Wang et al., 2020)]. **(A)** UMAP (Uniform Manifold Approximation and Projection) plot of delineated clusters of the combined data. Endothelial cells (EC) and Cardiomyocytes (CM) have been highlighted. **(B)** Cardiomyocyte, endothelial cell, endocardial cell, fibroblast, pericyte, smooth muscle cell, and cell cycle marker expression in each cluster. **(C)** The combined UMAP separated according to the three samples.

We compared the single cell transcriptome data of the co-cultured hiPS-CMs and hiPS-ECs to recently published scRNAseq data of human heart derived cells (Wang et al., 2020). The combined UMAP (HEL24.3 co-culture, HEL47.2 co-culture, and human cells) and the clusterwise gene expression of cardiomyocyte, endothelial cell, endocardial cell, fibroblast, pericyte, smooth muscle cell, and cell cycle markers is presented in Figures 2A,B. The hiPS-ECs clustered together with human heart ECs, and the hiPS-CMs clustered with human heart CMs (Figures 2B,C). In addition, a small number of hiPS-derived cells clustered with human SMC, pericyte/myofibroblast, and fibroblast-like cells (Figure 2C). The proliferating hiPS-ECs formed their own cluster. As expected, the hiPS-derived cells were more homogeneous compared to the cells derived from the adult heart, for example the hiPS-CMs concentrated mainly in one cluster (Cluster 1).

### Transcriptome Analysis of Single-Cell Clusters Identifies Subtypes of hiPS-CMs and hiPS-ECs

**FIGURE 3:**
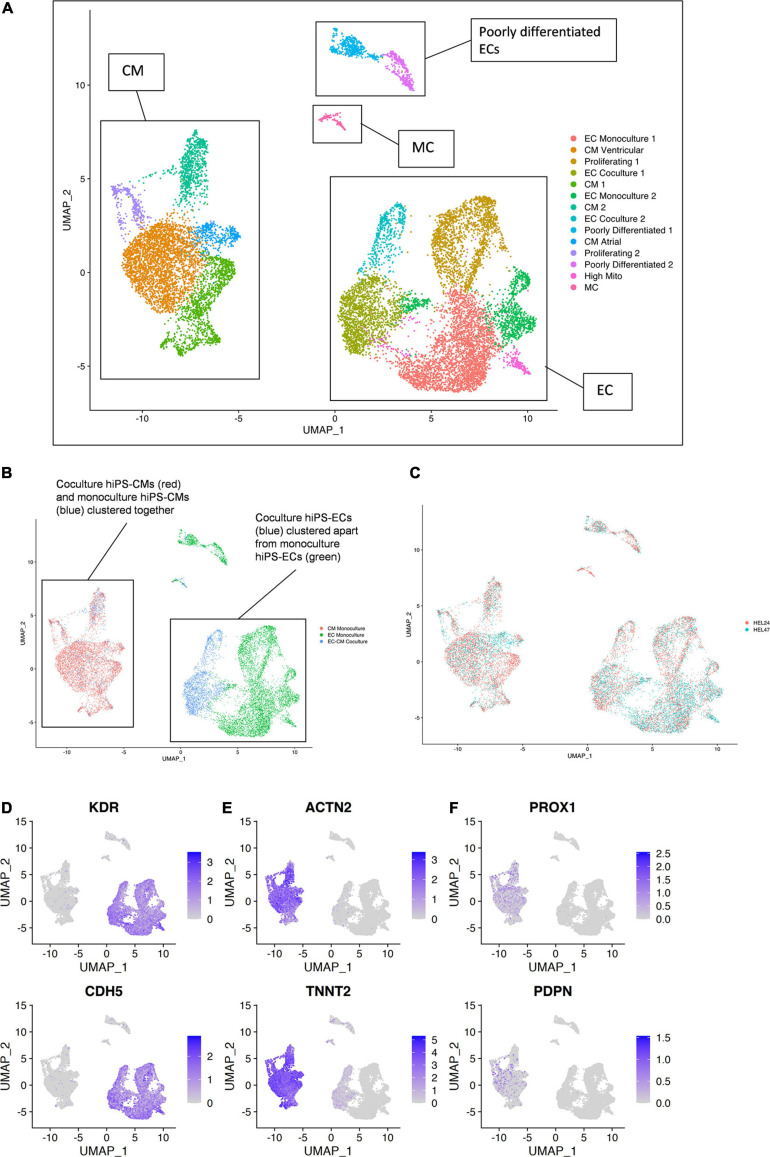
Single-cell transcriptome of six combined samples [(1) HEL24.3 hiPS-EC monoculture, (2) HEL24.3 hiPS-CM monoculture, (3) HEL24.3 co-culture of hiPS-CMs and hiPS-ECs, (4) HEL47.2 hiPS-EC monoculture, (5) HEL24.3 hiPS-CM monoculture, and (6) HEL47.2 co-culture of hiPS-CMs and hiPS-ECs]. **(A)** Uniform Manifold Approximation and Projection (UMAP) plot of delineated clusters. Endothelial cells (EC), cardiomyocytes (CM), mesenchymal cells (MC), and poorly differentiated ECs have been highlighted. **(B)** The same UMAP plot according to cell culture conditions [(1) hiPS-EC monoculture (green), (2) hiPS-CM monoculture (red), and (3) hiPS-EC/hiPS-CM co-culture (blue)]. **(C)** The same UMAP plot according to cell line (1) HEL24.3 (salmon), and (2) HEL47.2 (skyblue). Feature plots on the expression of **(D)** vascular endothelial markers **(E)** cardiomyocyte markers **(F)** lymphatic endothelial cell markers.

Single-cell RNA sequencing data from the six samples [(1) HEL24.3 hiPS-EC monoculture, (2) HEL24.3 hiPS-CM monoculture, (3) HEL24.3 co-culture of hiPS-CMs and hiPS-ECs, (4) HEL47.2 hiPS-EC monoculture, (5) HEL47.2 hiPS-CM monoculture, and (6) HEL47.2 co-culture of hiPS-CMs and hiPS-ECs] were combined for analysis. Clustering of the combined scRNAseq data resulted in 14 clusters (Figures 3A–C). HiPS-ECs formed separate, distinct clusters based on their growth conditions (monoculture vs. co-culture, Figure 3B). In contrast, mono- and co-culture hiPS-CMs clustered together (Figure 3B).

The hiPS-ECs had high expression of the endothelial cell markers *KDR* (85.8% of monoculture hiPS-ECs expressed) and *CDH5* (82.1%) (Figure 3D), and the hiPS-CMs had high expression of cardiomyocyte markers *ACTN2* (96.0% of monoculture hiPS-CMs expressed) and *TNNT2* (99.9%) (Figure 3E). The hiPS-ECs had very low, if any, expression of lymphatic EC markers *PROX1* and *PDPN* (Figure 3F). Low level expression of *PROX1* and *PDPN* was seen in some hiPS-CMs, as has been demonstrated previously (Petchey et al., 2014 and Human Protein Atlas).

**FIGURE 4:**
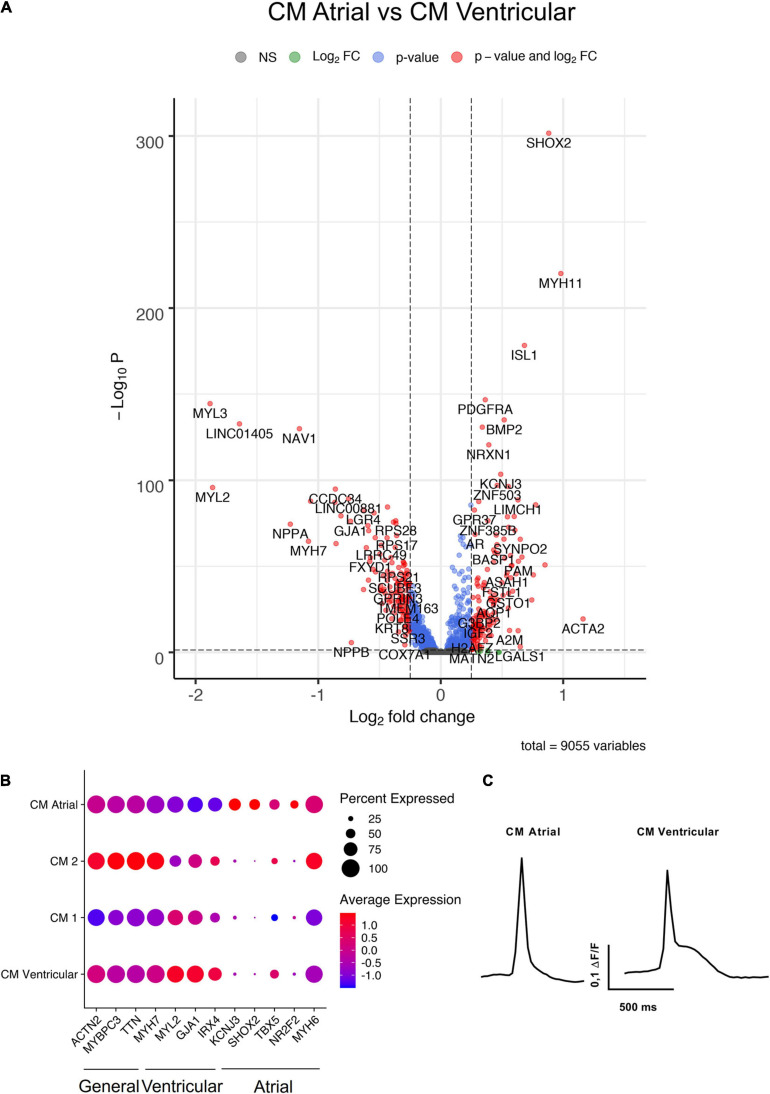
Characterization of hiPS-CM clusters. **(A)** Volcano plot of altered genes in atrial type hiPS-CMs (CM Atrial-cluster) compared to ventricular type hiPS-CMs (CM Ventricular-cluster). **(B)** Expression of general CM genes, atrial CM, and ventricular CM genes in the four hiPS-CM clusters. **(C)** Representative action potential morphologies of atrial and ventricular hiPS-CMs. All scRNAseq data presented in the figure consists of combined data from HEL47.2 and HEL24.3 lines.

Four of the hiPS-EC clusters consisted mainly of cells from monoculture (EC Monoculture 1 and 2, Proliferating 1 and High Mito), and two hiPS-EC clusters consisted mainly of co-culture cells (EC Co-culture 1 and 2). The hiPS-CMs formed five distinct clusters, of which one had an atrial (CM Atrial) and one a ventricular (CM Ventricular) gene expression profile (Figure 4A). In addition, there were two unspecific hiPS-CM clusters (CM Monoculture 1 and 2), and one cluster consisting of proliferating cells (Proliferating 2). The ventricular cluster had high expression of *MYL2*, *GJA1*, *MYH7*, and *IRX4* (Bao et al., 1999; Cui et al., 2019; Goldfracht et al., 2020), whereas the atrial cluster had high expression of *NR2F2*, *KCNJ3*, *TBX5*, and *SHOX2* (Devalla et al., 2015; Cui et al., 2019; Goldfracht et al., 2020; Figures 4A,B). Optogenetic analysis of the hiPS-CM contractility confirmed the presence of both atrial and ventricular cells with respective action potential features (Figure 4C).

In addition, there were 2 distinct clusters of cells with low EC gene expression, which we classified as poorly differentiated (Poorly differentiated 1 and 2), mainly consisting of monoculture hiPS-ECs. Finally, there was a very small cluster [Mesenchymal cells (MCs)], which expressed a mesenchymal cell phenotype with high expression of *ACTA2*, and *COL1A1*. Supplementary Figure 2A shows the contribution of each condition to the clusters highlighting that both poorly differentiated, and MC cluster cells likely originate from the hiPS-ECs even in the EC-CM Co-culture cluster, as these are nearly absent in the CM Monoculture cluster. Cells in the proliferating clusters (Proliferating 1 and 2) had high expression of cell cycle genes *MKI67*, *TOP2A*, and *BIRC5* (Supplementary Figure 2B).

#### Differences Between Differentiation of the HEL24.3 and HEL47.2 Cell Lines

The clustering was highly similar in the two independent (HEL24.3 and HEL47.2) cell lines (Figure 3C and Supplementary Figure 3). The CM Atrial cluster contained fewer cells in the HEL24.3 cell line, and instead a greater proportion of HEL24.3 cells formed the CM 1 cluster (Supplementary Figure 3B). HEL24.3 monoculture ECs also contained a larger proportion of proliferating cells compared to HEL47.2 (Supplementary Figure 3D).

### Co-culture Increased the Expression of Endocardial and Cardiac-Specific EC Genes in hiPS-ECs

**FIGURE 5:**
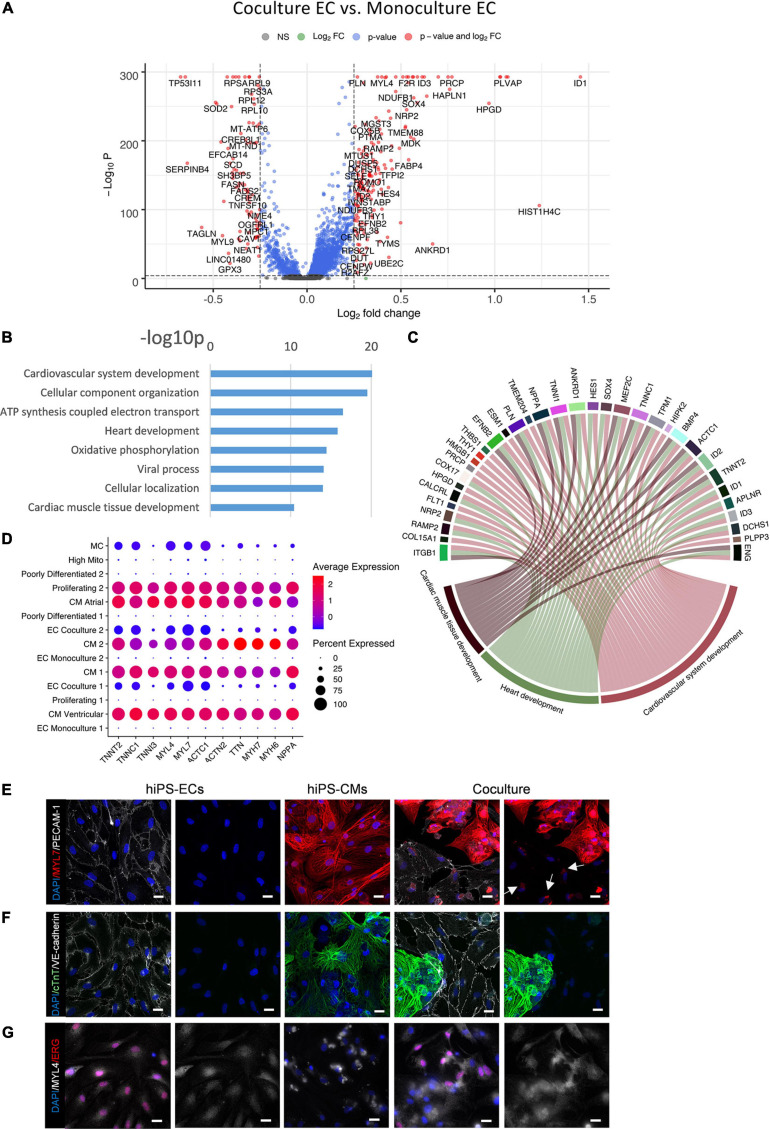
Comparison of co-culture and monoculture hiPS-ECs. **(A)** Volcano plot of altered genes in co-culture hiPS-ECs compared to monoculture hiPS-ECs. **(B)** GO-analysis on differentially expressed genes that are upregulated (log fold change > 0.10 fdr < 0.01) in co-culture hiPS-EC’s–eight representative categories are presented. **(C)** Upregulated genes in relevant GO categories (genes with log fold change > 0.25). **(D)** Expression of cardiac contractility genes and cardiac secreted protein NPPA coding gene in all clusters. **(E)** Immunofluorescence staining of MYL7 in monoculture hiPS-ECs, monoculture hiPS-CMs, and hiPS-EC/hiPS-CM co-culture (scale bar 20 μm). **(F)** Immunofluorescence staining of cTnT in monoculture hiPS-ECs, monoculture hiPS-CMs, and hiPS-EC/hiPS-CM co-culture (scale bar 20 μm). **(G)** Immunofluorescence staining of MYL4 in monoculture hiPS-ECs, monoculture hiPS-CMs, and hiPS-EC/hiPS-CM co-culture (scale bar 20 μm). All scRNAseq data presented in the figure consists of combined data from HEL47.2 and HEL24.3 lines. White arrows indicate MYL7 staining in hiPS-ECs.

Differential gene expression analysis revealed a profound effect of co-culture on the hiPS-EC transcriptome (Figure 5A). The significantly up- and downregulated genes for both cell types are presented in Supplementary Table 1.

A gene ontology classification (GO) analysis of the most significantly upregulated genes (adjusted *P* < 0.01 and average log fold change > 0.10) in hiPS-ECs in co-culture revealed activation of pathways associated with cardiovascular system and heart development, cellular component organization and oxidative phosphorylation (Figures 5B,C). More specifically, increased expression of several genes associated with heart development (*SOX4*, *NRP2*, *MEF2C*, *ENG*, *CALCRL*, *RAMP2*, *ANKRD1*, *ZFP36L1*, *DCHS1*, and *ENG*) (Dackor et al., 2006; Wirrig et al., 2007; Paul et al., 2014; Deshwar et al., 2016; Kechele et al., 2016; Materna et al., 2019; Figure 5C and Supplementary Figure 4A), cardiac endothelial-specific genes (*SPRX*, *FHL1*, and *IL6ST*) (Marcu et al., 2018), and endocardial genes (*NPR3*, *HAPLN1*, *PLVAP*, and *HEY2*) (Guo et al., 2016; Marcu et al., 2018; Miao et al., 2020; Supplementary Figure 4B) were observed in hiPS-ECs cultured together with hiPS-CMs. Interestingly, the presence of hiPS-CMs induced expression of cardiac myofibrillar contractility genes, such as *TNNT2*, *TNNC1*, *TNNI3*, *MYL4*, *MYL7*, *MYH6*, and *MYH7*, and the cardiac secreted protein *NPPA*, in the hiPS-ECs (Figure 5D), a phenomenon, which has been recently described also in isolated ECs from murine heart (Tabula Muris Consortium et al., 2018; Jambusaria et al., 2020; Paik et al., 2020; Yucel et al., 2020).

The expression of several junctional protein genes and their regulators were increased in hiPS-ECs in co-culture (Supplementary Table 1 and Supplementary Figure 4C). These included genes coding for adherens junction related proteins [*CDH5* (VE-cadherin), Catenin alpha-1 (*CTNNA1*) Catenin beta-1 (*CTNNB1*), and the tyrosine phosphatase VE-PTP (*PTPRB*) (Harris and Tepass, 2010; Nawroth et al., 2002)], tight junction related proteins [*ESAM1* (Nasdala et al., 2002) and *CLDND1* (Mineta et al., 2011)], and gap junction proteins Cx40 (*GJA5*) and Cx45 (*GJC1*) (Hautefort et al., 2019). In addition, the expression *TIE1* and *TEK* (*TIE2*), which have important roles in angiogenesis, vascular remodeling and endothelial barrier function (Savant et al., 2015; Komarova et al., 2017) were induced in co-culture hiPS-ECs.

### Myofibrillar Proteins MYL7 and MYL4 Are Expressed in Co-culture hiPS-ECs

To examine if the myofibrillar genes are also expressed at protein level, we used immunofluorescence staining for three myofibrillar proteins MYL7, MYL4 and cTnT, which were increased at the mRNA level in co-culture ECs by average log fold change 1.1, 0.4, and 0.5, respectively (all adjusted *p* < 1e-300). The stainings revealed the presence of small collections of MYL7 in co-culture hiPS-ECs close to the nucleus, which were absent in monoculture hiPS-ECs (Figure 5E). MYL4 staining was diffuse in the monoculture hiPS-ECs, while it was slightly stronger in co-culture hiPS-ECs (Figure 5F). In contrast, cTnT was not found in either co-culture or monoculture hiPS-ECs (Figure 5G). Since Alpha-actinin (Figure 1C) and cTnT were not seen in co-culture hiPS-ECs, we interpret this positive MYL7 and MYL4 staining to represent real protein expression and not for example remnants of hiPSCMs.

### HiPS-CM Transcriptome Was Less Affected by Co-culture

**FIGURE 6:**
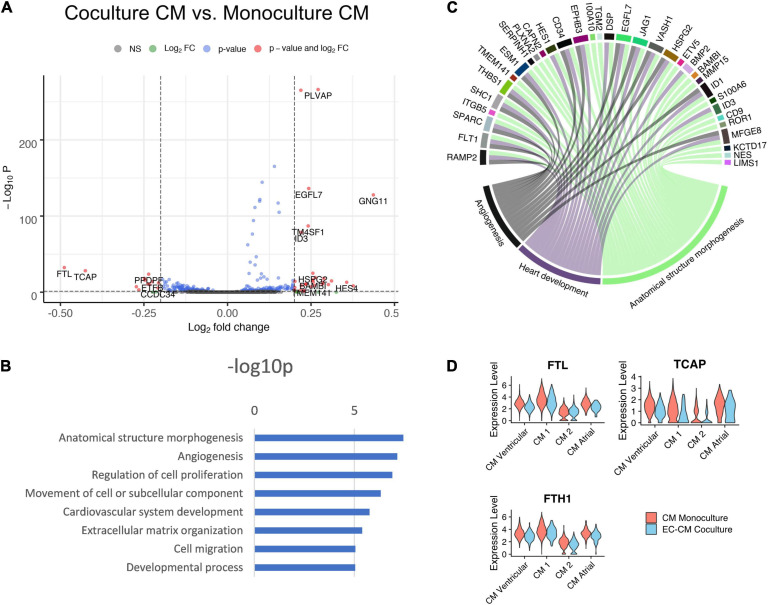
Comparison of co-culture and monoculture hiPS-CMs. **(A)** Volcano plot of altered genes in co-culture hiPS-CMs compared to monoculture hiPS-CMs. **(B)** GO-analysis on differentially expressed genes that are upregulated (fold change > 0.10) in co-culture hiPS-CMs–eight representative categories are presented. **(C)** Upregulated genes in three relevant GO categories. **(D)** The sarcomeric gene *TCAP*, and *FTH1* and *FTL* that build the cytoplasmic iron storage protein ferritin, were all significantly downregulated in co-culture hiPS-CMs. All scRNAseq data presented in the figure consists of combined data from HEL47.2 and HEL24.3 lines.

The effect of co-culture was less profound in the hiPS-CMs than in the hiPS-ECs (Figure 6A). According to GO analysis, the most upregulated pathways included anatomical structure morphogenesis, angiogenesis, regulation of cell proliferation and cardiovascular system development (Figures 6B,C). A sarcomeric gene *TCAP*, a load-sensitive regulator of t-tubule structure and function, was among the most downregulated genes in co-culture hiPS-CMs. In addition, *FTH1*, and *FTL* that build the cytoplasmic iron storage protein ferritin, were both significantly downregulated in co-culture (Figure 6D and Supplementary Table 1).

### Ligand-Receptor-Target Analysis Demonstrated Increased BMP-, Notch-, ErbB-, and VEGF-Signaling in Co-culture

**FIGURE 7:**
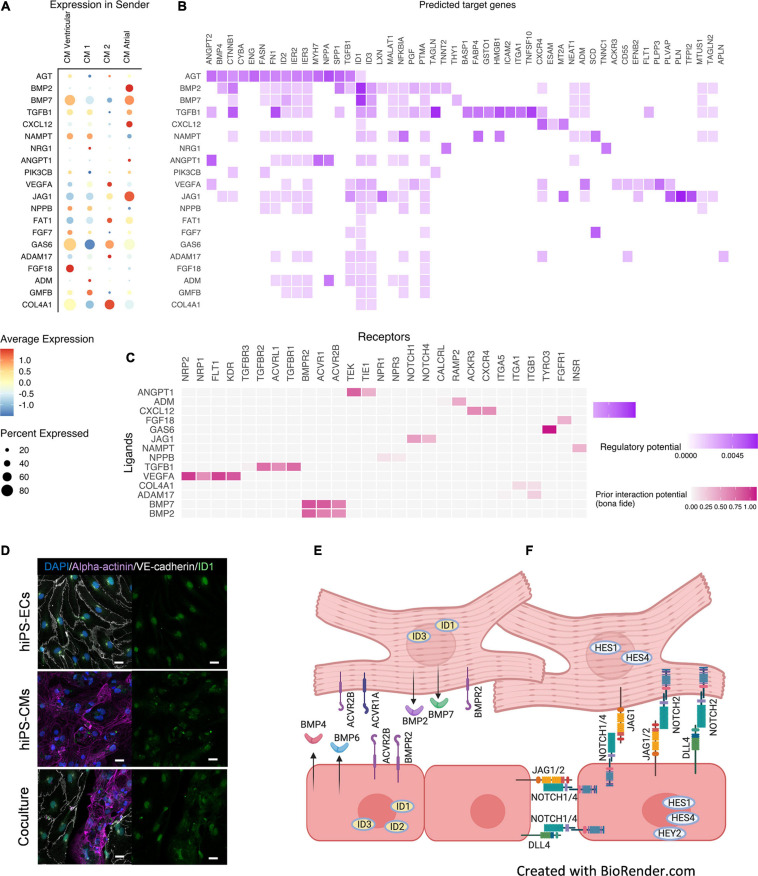
Ligand-target gene analysis using NicheNet (Browaeys et al., 2020). Results are shown for the top 20 ligands. **(A)** Expression of top 20 ligands in sender cells (hiPS-CM). **(B)** Ligand–target matrix denoting the regulatory potential between sender (hiPS-CM) ligands and predicted receiver (hiPS-EC) target genes. **(C)** Ligand-receptor interactions between ligands from hiPS-CMs and receptors expressed from hiPS-ECs. **(D)** Immunofluorescence staining of ID1 in monoculture hiPS-ECs, monoculture hiPS-CMs, and hiPS-EC/hiPS-CM co-culture (scale bar 20 μm). Predicted cell-cell interactions in co-culture hiPS-EC/hiPS-CM in **(E)** BMP-pathway, where increased expression of BMP2/7 ligands is seen in hiPS-CMs and BMP4/6 in hiPS-ECs in co-culture as well as an increased expression of the ID-genes in both cell types, **(F)** Notch-pathway, where the ligand JAG1 is increased in hiPS-ECs and hiPS-CMs and JAG2 and DLL4 in hiPS-ECs, in co-culture resulting in an increased expression of the targets HES1 and HES4 in hiPS-CMs, and HES1, HES4, HEY2 in hiPS-ECs (Data in Supplementary Figure 5). All scRNAseq data presented in the figure consists of combined data from HEL47.2 and HEL24.3 lines.

A ligand-receptor-target analysis NicheNet (Browaeys et al., 2020), where the hiPS-CMs were defined as a sender cell type and hiPS-ECs as receiver, demonstrated enhanced BMP-, Notch-, and VEGF-signaling (Figures 7A–C and Supplementary Figure 5). BMP-signaling is an important regulator of cardiac development, and we observed strongly increased expression of BMP-pathway ligands and targets in both hiPS-ECs and hiPS-CMs in co-culture. Changes in some of the BMP-pathway receptors were also observed. The hiPS-CMs express high levels of BMP-ligands *BMP2*, *BMP5*, and *BMP7*, whereas the expression is low in hiPS-ECs. Thus, the hiPS-ECs were exposed to significantly higher amounts of these ligands in co-culture than in monoculture (Figure 7A and Supplementary Figure 5A), which is reflected by markedly increased expression of BMP-targets *ID1*, *ID2*, and *ID3* genes in hiPS-ECs (Supplementary Figure 5C and Supplementary Table 1). *ID1* and *ID3* were also slightly upregulated in the co-culture hiPS-CMs compared to monoculture hiPS-CMs (Supplementary Table 1). Interestingly, atrial type hiPS-CMs had significantly higher *BMP2* expression compared to the other hiPS-CMs (Supplementary Figure 5A). In addition, the expression of *BMP4* and *BMP6* increased significantly in the co-culture hIPS-ECs (Supplementary Figure 5A and Supplementary Table 1). Increased levels of *BMPR2* expression, and also low but increased levels of *ACVR1* and *ACVR2B* expression were observed in co-culture hiPS-ECs compared to monoculture hiPS-ECs (Supplementary Figure 5B and Supplementary Table 1). Interestingly, the expression of the decoy receptor *BAMBI* increased in both cell types in co-culture (Supplementary Table 1). Immunofluorescence staining of ID1 revealed that in hiPS-ECs it was highly restricted to the nucleus whereas it was more diffuse in hiPS-CMs (Figure 7D).

Notch-ErbB signaling between endocardium and myocardium is essential during cardiogenesis, especially in myocardial trabeculation (Del Monte-Nieto et al., 2018). NicheNet -analysis indicated increased Notch-signaling in both cell types in co-culture compared to the monocultures (Figures 7B,C). Thus, we examined the effect of co-culture on the expression of the Notch and ErbB-pathway mediators in more detail. The Notch-ligand *JAG1* was expressed in the hiPS-CMs, and low levels of *JAG1* were also detected in the hiPS-ECs. In both cell types the expression levels significantly increased in co-culture (Supplementary Figure 5A and Supplementary Table 1). In addition, expression of the Notch-ligands *JAG2* and *DLL4* increased in co-culture hiPS-ECs (Supplementary Figure 5A and Supplementary Table 1). A marked increase in the expression of the Notch-receptors *NOTCH1* and *NOTCH4* was observed in co-culture hiPS-ECs (Supplementary Figure 5B and Supplementary Table 1). Of the Notch-pathway targets, *HES1*, *HES4*, and *HEY2* expression levels were higher in the co-culture hiPS-ECs compared to monoculture cells (Supplementary Figure 5C and Supplementary Table 1), and *HES1* and *HES4* expression was higher in the co-culture hiPS-CMs as compared to monoculture cells (Supplementary Table 1). This indicates bidirectional Notch pathway activation in both cell types upon interaction with the other cell type.

Increased expression of VEGF-receptors were seen in co-culture hiPS-ECs (Supplementary Figure 5B), possibly mediated by the VEGFA and VEGFB expression in the hiPS-CMs.

We repeated the NicheNet analysis changing hiPS-ECs to sender cells and hiPS-CMs to receiver cells (Supplementary Figure 6). Besides the BMP- and Notch-pathways, the analysis indicated alterations in the ErbB-signaling pathway. Further analysis of the ErbB-pathway ligands showed that *HB-EGF* was highly expressed in hiPS-ECs in both conditions, and *NRG1* was induced in co-culture (Supplementary Figure 7 and Supplementary Table 1), whereas the expression of the other ligands (*NRG2*, *NRG3*, *NRG4*, *AREG*, *BTC*, *EREG*, *EPGN*, and *TGFA*) were low. Of the ErbB-receptors, *ERBB2* and *ERBB4* were highly expressed in hiPS-CMs in both monoculture and co-culture, while the expression levels of *EGFR* and *ERBB3* were low (Supplementary Figure 7). In the hiPS-ECs, ErbB receptor expression was low in all conditions (Supplementary Figure 7). The expression of *ERBB2* interacting protein *ERBB2IP* (ERBIN), which is important for *ERBB2* stabilization and *NRG1* signaling (Tao et al., 2009) and is considered as a negative modulator of pathological cardiac hypertrophy (Rachmin et al., 2014), was expressed in both cell types, and increased in co-culture hiPS-ECs (Supplementary Figure 7 and Supplementary Table 1).

A schematic showing the predicted effects of co-culture on BMP- and Notch-pathway and the clusterwise expression of BMP-, Notch-, and ErbB-pathway genes in both independent cell lines are shown in Figures 7E,F and Supplementary Figure 8.

### Validation of the Co-culture Effects on hiPS-ECs by an Independent Experiment

To replicate the effects of the hiPS-CMs on the hiPS-ECs, we performed an independent scRNAseq experiment consisting of hiPS-EC/CM co-culture samples from six individual cell lines pooled together. The UMAP of the pooled cells is presented in Supplementary Figure 9A. The expression of cardiac contractility genes and cardiac secreted protein NPPA coding gene in hiPS-ECs (Supplementary Figure 9B, the same genes as in Figure 5D) were also observed in this data set as well as high expression of genes related to heart development and endocardial genes (Supplementary Figure 9C, the same genes as presented in Supplementary Figures 4A,B). Also high expression of BMP-, Notch- and ErbB-pathway genes was observed in this data, indicating the reproducibility of our findings (Supplementary Figure 9D, the same genes as presented in Supplementary Figures 8A–C).

## Discussion

Endothelial cells and cardiomyocytes communicate with each other by cardiokine and angiocrine signaling, and this crosstalk has an essential role in cardiac development, growth, and homeostasis. Here we show that the hiPS-EC transcriptomic landscape is strongly affected by the presence of hiPS-CMs, resembling organotypic adaptation to the requirements of the parenchymal cells. This is reflected by augmented expression of genes important for cardiac development, and genes that have organotypic specificity in cardiac ECs and the endocardium. Interestingly, hiPS-ECs in co-culture expressed myofibrillar contractile genes, of which most were not observed in monoculture. Further, single-cell RNA sequencing revealed distinct transcriptomic subpopulations in both cell types and identified BMP-, Notch-, and ErbB-signaling pathways and their components as mediators of the crosstalk. Since both cell types stayed rather segregated in co-culture, with limited physical contact with each other, it is likely that a majority of the effect is mediated by secreted factors. Our results also highlight the importance of single-cell RNA sequencing to distinguish different phenotypes of hiPSC-derived ECs and CMs and the potential of EC-CM co-culture in modeling of organotypic developmental processes and cardiac diseases on a dish.

Exposure to hiPS-CMs induced expression of several cardiomyocyte contractility genes in the hiPS-ECs. Expression of contractile cardiac genes has been recently described in murine heart derived ECs (Tabula Muris Consortium et al., 2018; Jambusaria et al., 2020; Yucel et al., 2020). This expression has been speculated to occur as adaptation of the cardiac endothelium to the cardiokine signaling of the surrounding cardiomyocytes (Jambusaria et al., 2020). Another offered explanation for this phenomenon has been mRNA contamination from cardiomyocytes during cell isolation. However, Yucel et al. (2020) recently elegantly showed that cardiomyocyte myofibril mRNAs originate in ECs, via active maintenance of open chromatin and transcription. This finding is recapitulated in our co-culture human iPS-ECs already after a relatively short (48 h) exposure to hiPS-CMs, demonstrating the magnitude of the crosstalk between these two cell types. Yucel et al. (2020) showed that, at any given time, only a subset of cardiac ECs express only few cardiomyocyte myofibril genes, suggesting stochastic expression triggered by paracrine question from CMs. This is in accordance with our data, as the expression was seen only in a subset of co-culture hiPS-ECs, and the expression levels were clearly lower than in hiPS-CMs. Yucel et al. (2020) also proposed that as CMs and cardiac ECs have a shared developmental origin, the accessibility of chromatin at cardiomyocyte-specific genes might be due to epigenetic memory. However, our findings perhaps support more the direct effect of paracrine crosstalk, since the finding was recapitulated in a much more simplified system compared to *in vivo* heart development. Our results also expand this finding to human cells.

Thus far it has been unclear if the cardiomyocyte contractile gene mRNA expression results in protein translation in cardiac ECs. Immunofluorescence staining of co-culture hiPS-ECs revealed expression of MYL7 as small collections near the nuclei, which were not observed in the monoculture hiPS-ECs. Low expression levels of MYL4 were seen in monoculture hiPS-ECs, and the staining was enhanced in the co-culture hiPS-ECs. Expression of *Myl4* and *Myl7* genes has also been documented murine heart derived ECs in Tabula Muris Consortium et al. (2018), which is in accordance with our results. Expression of cTnT (TNNT2) or ACTN2 was not detected in either co-culture or monoculture hiPS-ECs, indicating that all cardiomyocyte contractile genes that are expressed as mRNA do not lead to observable levels of protein translation, at least continuously. The lack of presence of TNNT2 or ACTN2 proteins also speak against remnants of dying hiPS-CM causing the MYL4 and MYL7 protein expression in co-culture hiPS-ECs, and support real protein expression by hiPS-ECs themselves. The presence and functional role of this selective protein expression in co-culture hiPS-ECs remains to be further elucidated. In addition, it is currently unknown if a similar phenomenon occurs *in vivo*.

The fast acquisition of organotypic EC identity of the hiPS-ECs in co-culture was demonstrated by the augmented expression of several genes associated with heart development (Dackor et al., 2006; Wirrig et al., 2007; Kinderlerer et al., 2008; Kern et al., 2010; Deshwar et al., 2016; Kechele et al., 2016; Materna et al., 2019) and expression of specific cardiac endothelial cell genes (Marcu et al., 2018). Among the most upregulated genes were e.g., *HAPLN1* and *ID1*, both important regulators of ECM in the developing heart (DeLaughter et al., 2013, 2016).

Many of the co-culture-induced genes also contribute to vascular development and angiogenesis, reflecting the response to angiogenic factors produced by cardiomyocytes. Interestingly, genes related to oxidative phosphorylation were upregulated in co-culture hiPS-ECs and *HIF1A*, *GAPDH*, and *LDHA* were downregulated, suggesting that aerobic metabolism is promoted in ECs, when co-cultured with CMs. Similarly to the hiPS-ECs, increased expression of genes associated with cardiovascular development was seen in co-culture hiPS-CMs, although the overall effect of co-culture was less pronounced in the hiPS-CMs. Moreover, upregulation of several angiogenic genes were detected in hiPS-CMs and their receptors in hiPS-ECs, reflecting enhanced angiogenic signaling from CM to EC via cardiokines (e.g., VEGFA and VEGFB). Interestingly, several junctional protein genes were upregulated in hiPS-ECs in co-culture, potentially indicating that the presence of hiPS-CMs led to improved endothelial barrier function, cellular crosstalk and stability (Komarova et al., 2017; Hautefort et al., 2019). We also observed a similar effect in flow-exposed hiPS-ECs (Helle et al., 2020).

Ligand-receptor analysis revealed activation of signaling pathways previously shown to mediate EC-CM crosstalk during cardiac development and growth in mice. Co-culture significantly affected the expression of several BMP and Notch-pathway ligands, receptors, targets, and effectors in both cell types in co-culture. While continuous BMP-signaling is required during early cardiogenesis, during later development atrial and ventricular cardiomyocytes require BMP signaling at different stages (de Pater et al., 2012). Interestingly, we observed differences in atrial and ventricular hiPS-CMs in BMP-signaling. For example, *BMP2* was mainly expressed in atrial-type hiPS-CMs, and *BMP5* and *BMP7* were highly expressed in both atrial and ventricular type cells. In addition, the expression levels of the decoy receptor *BAMBI* were highest in atrial-type cardiomyocytes. These examples demonstrate the heterogeneity of these cells, and possibly indicate a cell-type specific regulatory effect of hiPS-ECs on hiPS-CM BMP-signaling. Markedly increased levels of ID-genes in both hiPS-ECs and hiPS-CMs in co-culture further underline the strong effect these cells have on each other.

Notch-signaling has an important role in the patterning of the early embryonic endocardium to valve and chamber formation, and later regulating the outflow tract and valve morphogenesis (MacGrogan et al., 2016). Notch and ErbB mediated EC-CM crosstalk is essential in the development of ventricular trabeculae (D’Amato et al., 2016). Increased expression of several Notch-pathway ligands and targets in both cell types demonstrates the activation of this pathway in co-culture. The expression of Notch ligands *JAG1* and *DLL4* in hiPS-ECs, and the higher expression of *JAG1* in hiPS-CMs in co-culture resembled that of early phases of cardiogenesis in mice (Luxán et al., 2016). Interestingly, the expression of *JAG1* was higher in the atrial-like hiPS-CMs, which is consistent with the gene expression pattern in murine embryonic hearts (Loomes et al., 1999). The general expression pattern of Notch ligands and targets also resemble that of the adult human heart where JAG1 in the parenchymal cells interact with endothelial NOTCH1 and NOTCH4, and JAG1, JAG2 and DLL4 in the endothelial cells interact with NOTCH2 and NOTCH3 in parenchymal cells (Litviňuková et al., 2020).

Interestingly, an endocardial marker Plasmalemma vesicle-associated protein (*PLVAP*) was among the most upregulated genes in both cell types in co-culture. PLVAP is specific for endothelial cells and it forms the stomatal and fenestral diaphragms of blood vessels. Its functions include regulating basal permeability, leukocyte migration and angiogenesis in ECs (Guo et al., 2016), and it is upregulated in pathological angiogenesis (Li et al., 2019). In the murine heart, it is rather specific for endocardium and newly formed vessels, e.g., after myocardial infarction (Li et al., 2019). The role PLVAP in EC-CM crosstalk remains to be further defined.

Based on both the scRNAseq clustering and the number of differentially expressed genes, the hiPS-EC transcriptome was largely affected by the presence of cardiomyocytes, whereas the hiPS-CMs was affected more modestly. This demonstrates the plasticity of ECs similarly to what is observed *in vivo*, where ECs adapt to their environment by acquiring organotypic features. It also reflects the developmental state of the hiPS-ECs with high plasticity in response to paracrine signals. However, although the hiPS-CMs are considered to resemble embryonic cardiomyocytes, they had a markedly smaller response to co-culture than the hiPS-ECs.

As expected, the basic gene expression profiles or responses to stimulation were not fully identical in the two studied cell lines. However, considering the cell line differences, the major effects described here were replicated well in both cell lines and in a separate scRNAseq validation experiment. The clustering was similar in both hiPS-EC cell lines as the co-culture hiPS-ECs clearly formed their own cluster separate from the static monoculture hiPS-ECs. In addition, both cell lines had cells consisting mainly of static monoculture hiPS-ECs that had lower expression of EC markers, and thus defined as poorly differentiated. In co-culture, these cells were not present, indicating the maturation effect of CMs on ECs. An interesting observation in the hiPS-CMs was that the Atrial CM cluster was rather small in the HEL24.3 cell line compared to HEL47.2 cell line, and the HEL24.3 cell line had a larger unspecific CM cluster that resembled more ventricular cells, but also had some atrial gene expression. One possible explanation could be the maturity of the hiPS-CMs, as the HEL24.3 were younger at the time of the experiment, thus, the intermediate cluster could represent a more naive cluster. It could be plausible that the differentiation into atrial and ventricular cells occurs when more time is given for the cells to mature. One hiPS-CM differentiation protocol has been shown to lead to decreased number of atrial hiPS-CMs and increased number of ventricular hiPS-CMs as the differentiation advanced (Burridge et al., 2014), which is in contrast with our speculation. As we did not analyze the proportion of hiPS-CM subtypes during the differentiation, our study cannot confirm or deflate these results. In any case, the heterogeneity of hiPS-CMs detected by scRNA sequencing is an important observation, which should be taken into consideration, when comparing hiPS-CMs from different individuals and conditions. Moreover, when comparing the single cell transcriptome data of the co-cultured hiPS-CMs and hiPS-ECs to scRNAseq data of human heart derived cells, it was seen that although the hiPS-ECs clustered together with human heart ECs and hiPS-CMs clustered together with human heart CMs, the hiPSC derived cells, especially hiPS-CMs, were clearly more homogenous than those of the human heart. This demonstrates that tissue specific hiPSCs are still rough simplifications of those seen *in vivo*.

Our study sheds light on the interactions and transcriptomic changes induced by endothelial cell-cardiomyocyte crosstalk, which is an important regulator of cardiac development and adult cardiac homeostasis in health and disease. hiPSC-derived endothelial cells and cardiomyocytes provide an excellent translational model to study organotypic development of cardiac vasculature and the EC-CM interactions. The transcriptomic changes observed in the co-culture cells after 48 h demonstrate the importance and magnitude of this signaling. Moreover, the extent of phenotypic change in the hiPS-ECs suggests that striving toward more physiological conditions in cell culture could provide us with more accurate disease models. Finally, the observation of atrial and ventricular hiPS-CM subclusters demonstrates the value of using scRNAseq, as specific cell types clearly respond differently to the stimuli.

## Experimental Procedures

### Data Availability

The scRNAseq datasets generated for this study are deposited in Gene Expression Omnibus (GEO) database with accession number GSE150741.

### hiPS Cell Lines

Two hiPSC lines (HEL47.2, HEL24.3) were obtained from The Biomedicum Stem Cell Center Core Facility (University of Helsinki; https://www2.helsinki.fi/en/infrastructures/genome-editing-function-and-stem-cell-platform/infrastructures/biomedicum-stem-cell-center). The cell lines were created by using retroviral/Sendai virus transduction of Oct3/4, Sox2, Klf4, and c-Myc, as described previously (Trokovic et al., 2015a, b). hiPSCs were maintained in Essential 8 media (A1517001, Thermo Fisher Scientific) on thin-coated Matrigel (354277, dilution 1:200; Corning, Corning, NY, United States). The cells were passaged using EDTA.

### hiPS-EC Differentiation

Endothelial cell differentiation was conducted based on the protocol by Giacomelli et al. (2017). The BPEL medium ingredients were purchased from the same vendors as mentioned in the article, except for BSA (A7030, Sigma) and PVA (362607, Sigma). Briefly, 125,000–175,000 cells/well in a six-well plate were plated on day 0. On day 1, the medium was changed to BPEL with 20 ng/ml BMP4 (120-05ET, PeproTech), 20 ng/ml Activin A (AF-120-14E-50ug, PeproTech) and 4 μmol/L CHIR (S2924, Selleckchem). On day 3, the medium was changed to BPEL with 50 ng/ml VEGF (produced in-house) and 5 μmol/L IWR-1 (I0161, Sigma). On day 6, medium was changed to BPEL with 50 ng/ml VEGF and the cells were maintained in this medium until they were sorted (day 7–9). 50 ng/ml VEGF was maintained in all cell culture with hiPS-ECs unless otherwise indicated.

### hiPS-EC Sorting

After differentiation, hiPS-ECs were sorted using magnetic beads with an antibody against CD31 (130-091-935, Miltenyi Biotec), according to the manufacturer’s protocol. The concentration of the cells was counted with Bio-Rad TC10 or TC20 Automated Cell Counter. The cells were immediately used for experiments.

### hiPS-CM Differentiation

hiPSC-derived cardiomyocytes differentiation was modified from Sharma et al. (2015). hiPS cells were plated 1:10 −1:15 in 12-well plates coated with Matrigel. After reaching confluency (day 0), differentiation was started by changing the media to RPMI (10-040-CV, Corning) and B27 supplement without insulin (A1895601, Thermo Fisher Scientific), with 5 μmol/L CHIR. The following day (day 1), the same media with CHIR was added to the cells. On day 2, the media was changed for RPMI/B27 without insulin, with 5 μmol/L IWR-1 (I0161, Sigma), and replenished on day 3. On day 4 and 5, RPMI/B27 without insulin was used. For days 6–8, the media was changed to RPMI media and B27 with insulin (17504044, Thermo Fisher Scientific). On day 9, RPMI without glucose (10-043-CV, Corning) and B27 with insulin, but with added lactate (L7900, Sigma, 466 μl/500ml) was used. The cells were split on day 13, 14, or 15, using RPMI media and B27 with insulin. The next day the media was changed to RPMI without glucose and the cells were maintained in this media until they were used in experiments. The media was changed to BPEL 2–3 days before the start of the experiments.

### qPCR Analysis of CM Marker Gene Expression

RNA samples were collected from CM differentiation experiments on days 1, 3, 5, 7, 9, 11, 13, and 20. RNA was extracted using Nucleospin RNA Plus Extraction kit (740984, Macherey-Nagel). cDNA synthesis was performed using High Capacity cDNA Reverse Transcription Kit (4368814, Thermo). qPCR analysis was done using SYBR Green (04913914001, Roche) and Bio-Rad CFX96 Real-Time PCR Detection System. All results were first normalized to housekeeping gene RPL37A and then to the expression levels during day 1.

### Preparation of hiPS-CMs for Optogenetics

For optogenetic analysis, lentiviral vector Optopatch [a kind gift from Adam E. Cohen group (Hochbaum et al., 2014; Dempsey et al., 2016) acquired through Addgene, plasmid # 62984], was introduced in hiPS-CMs, using 1 pg of virus per cell for transduction. During lentiviral work, the cells were cultured in 12-well plates coated with Matrigel and detached twice using Accutase to remove replicating virus. RPMI/B27 medium with glucose was used 48 h after transduction and both passages. Otherwise the cells were cultured in RPMI/B27 medium with lactate. On the second passage, the cells were plated on Matrigel-coated glass-bottom dishes (14 mm Ø, P35G-1.5-14-C, MatTek) for optogenetic imaging. William’s E medium (Life Technologies A12176) supplemented with Cocktail B (Life Technologies CM4000) without dexamethasone was used as imaging medium.

### Optogenetic Video Imaging of hiPS-CMs

Action potentials were recorded from spontaneously beating hiPS-CMs expressing Optopatch. Optogenetic imaging platform included environmental chamber (5% CO2, 37°C, EMBL), Nikon Eclipse Ti-E fluorescence microscope, red laser light source (λ = 647 nm, Ef = 550 mW/mm2) and NIS-Elements advanced research software for the platform operation. Raw data was recorded as image sequences (50 frames per second), from which the total fluorescence intensity signal was exported to MS Excel. Data was normalized by fitting the acquired signal to an exponential function with cPot Cardiac Action Potential Calculator software, written in MATLAB. The optogenetic imaging platform and workflow is described in more detail in Björk et al. (2017).

### hiPS-EC and hiPS-CM in Monoculture and in Co-culture

For co-culture, hiPS-CMs and hiPS-ECs were plated together in BPEL media with 50 ng/ml VEGF. Also the monoculture hiPS-ECs and hiPS-CMs were re-plated in BPEL media with 50 ng/ml VEGF at this time point to account for the potential transcriptomic differences resulting from the splitting. The concentration of cells was calculated using Bio-Rad TC10 or TC20 Automated Cell Counter. For scRNAseq experiments, 1 million hiPS-CMs and 200,000 ECs per well were plated together on a 12-well plate, and the same amount of hiPS-CMs and hiPS-ECs in monoculture were plated at the same time. After 48 h of co-culture or monoculture, the cells were analyzed by single-cell RNA sequencing (scRNAseq). hiPS-CM HEL47.2 cells were 41 days old, and HEL24.3 cells were 37 days old at the time of cell collection for single-cell RNA-seq, and the hiPS-ECs were used immediately after differentiation and sorting.

### Immunofluorescence Staining

Cells were fixed with 4% PFA and stained with monoclonal anti-α-Actinin (Sarcomeric) (A7811, Sigma), VE-cadherin (2500, Cell Signaling Technology), PECAM-1 (M0823, Dako), MYL7 (17283-1-AP, Proteintech), MYL4 (67533-1-Ig, Proteintech) and cTnT (MAB1874, R&D Systems) antibodies. Nuclei were visualized with DAPI. Stained cells were imaged with fluorescent or confocal microscopes (Zeiss AxioImager and Zeiss LSM 780).

### Processing of Cells for scRNAseq

The cells were detached using Accutase (A6964, Sigma) and their concentration was measured. The cells were washed once with PBS containing 0.04% BSA, and then resuspended in PBS with 0.04% BSA to a concentration of 0.79–1.0 × 10^6^ cells/ml. The cells were passed through a 35 μm strainer (352235, Corning) and placed on ice until continuation of the 10× Genomics Single Cell Protocol at the Institute of Molecular Medicine Finland (FIMM), where the concentration and viability of cells was calculated with Luna Automated Cell Counter and 4000 cells/sample were processed.

### Single-Cell RNA Sequencing

Single-cell gene expression profiles were studied using the 10× Genomics Chromium Single Cell 3′RNAseq platform. The Chromium Single Cell 30 RNAseq run and library preparation were done using the Chromium Single Cell 30 Reagent version 2 chemistry. The sample libraries were sequenced on Illumina NovaSeq 6000 system. 4000 cells and 50,000 PE/cell were analyzed. Data processing and analysis were performed using 10 × Genomics Cell Ranger v2.1.1 pipelines. The “cellranger mkfastq” pipeline was used to produce FASTQ (raw data) files. The “cellranger count” was used to perform alignment, filtering and UMI counting. mkfastq was run using the Illumina bcl2fastq v2.2.0 and alignment was done against human genome GRCh38. Cellranger aggr pipeline was used to combine data from multiple samples into an experiment-wide gene-barcode matrix and analysis.

Analyses were performed with Seurat R package version 3.2.1 (Stuart et al., 2019). The standard preprocessing (log-normalization) was done using the NormalizeData function and variable features were identified individually for each using the FindVariableFeatures function. Cells, in which a minimum of 1000 and maximum of 6000 genes were detected, were included, and cells with a mitochondrial gene content of >30% were excluded. The FindIntegrationAnchors function was used to identify anchors, which were passed to the IntegrateData function to create a batch-corrected expression matrix enabling the two datasets to be jointly analyzed. Principal component analysis (PCA) was then performed on the highly variable genes. The first 30 PCs were used for uniform manifold approximation (UMAP). Differential expression for each subpopulation in the scRNAseq data was performed using the FindAllMarkers function (Wilcoxon Rank Sum test) in Seurat, and FindMarkers was used to distinguish different conditions. Cells were clustered based on their expression profile setting the resolution to 0.3, which led to 13 clusters which were quite clearly distinguished in the UMAP. Established markers on The Human Protein Atlas and published literature were used to annotate cell types.

### Single-Cell RNA Sequencing Data Comparison Between hiPSC and Normal Human Heart

Analyses for the dataset comparison were performed using R-package Seurat version 3.2.1 for the three different datasets [HEL24.3 hiPS-CM/hiPS-EC co-culture, HEL47.2 hiPS-CM/hiPS-EC co-culture, and normal human heart GSE109816 (Wang et al., 2020)]. Preprocessing was done using the function “SCTransform,” which normalizes and scales the data in addition to finding variable features. Cells with a minimum of 1000 features, maximum of 7000 features and <80% of mitochondrial content were included. The effect of cell cycle and mitochondrial genes were regressed out using cell cycle scoring. The three datasets were combined using Seurat functions “SelectIntegrationFeatures,” “PrepSCTIntegration,” “FindIntegrationAnchors,” and “IntegrateData” using canonical correlation analysis as a reduction method. PCA and then UMAP were performed as a dimensional reduction method using 30 dimensions from PCA. Clusters were formed with Seurat functions “FindNeighbors” and “FindClusters” using resolution of 0.5. This formed 13 clusters, which were visualized using the function “DimPlot.” “DotPlot” was used with normalized RNA assay to identify clusters and cell type markers were identified using published literature.

### Ligand-Receptor Analysis

The ligand-receptor analysis was done using the R package NicheNet (Browaeys et al., 2020) to explore cell-to-cell communication between hiPS-ECs and hiPS-CMs. Co-culture hiPS-CMs were defined as “sender” cells and co-culture hiPS-ECs as “receiver” cells and vice versa. For the “receiver” cells top 20 ligands were selected to predict the responses.

### Gene Ontology-Analysis

The gene ontology (GO)-analysis was done using the Database for Annotation, Visualization and Integrated Discovery (DAVID) v6.8. Only genes with a log fold change > 0.25 were included.

## Data Availability Statement

The datasets presented in this study can be found in online repositories. The names of the repository/repositories and accession number(s) can be found in the article/Supplementary Material.

## Ethics Statement

The studies involving human participants were reviewed and approved by Coordinating Ethics Committee of the HUS Hospital District.

## Author Contributions

EH, RK, and AD: conceptualization. EH: data curation and project administration. EH, ETe, and ETo: formal analysis and software. EH and RK: funding acquisition and supervision. MA, EH, LA, and ETe: investigation. EH, MA, ETe, and EM: methodology. EH, RK, and EM: resources. EH, MA, and ETe: visualization. EH and MA: writing—original draft. RK, AD, and EM: writing—review and editing. All authors contributed to the article and approved the submitted version.

## Conflict of Interest

The authors declare that the research was conducted in the absence of any commercial or financial relationships that could be construed as a potential conflict of interest.

## Publisher’s Note

All claims expressed in this article are solely those of the authors and do not necessarily represent those of their affiliated organizations, or those of the publisher, the editors and the reviewers. Any product that may be evaluated in this article, or claim that may be made by its manufacturer, is not guaranteed or endorsed by the publisher.

## Supplementary Material

Supplementary Figure 1 Expression of cardiomyocyte markers during differentiation. qPCR analysis shows that CM marker gene expression is increased during differentiation. Two differentiation experiments [HEL47.2 (1.) and HEL47.2 (2.)] with two replicates each are shown. Expression levels have been normalized by comparing to day 1.

Supplementary Figure 2 **(A)** UMAP split according to sample (CM Monoculture, EC Monoculture, and EC-CM Co-culture. **(B)** EC, CM, cell cycle and mesenchymal cell gene expression in the defined clusters. All scRNAseq data presented in the figure consists of combined data from HEL47.2 and HEL24.3 lines.

Supplementary Figure 3 Comparison of hiPS transcriptomics between HEL47.2 and HEL24.3 lines. **(A)** Samplewise distribution of cells per cluster. Distribution of cells per cell type cluster according to sample in **(B)** hiPS-CM, **(C)** hiPS-EC in co-culture, **(D)** hiPS-EC in monoculture.

Supplementary Figure 4 Co-culture vs. monoculture hiPS-EC expression of **(A)** genes related to heart development, **(B)** cardiac EC genes, and **(C)** genes coding for junctional proteins and their regulators. All scRNAseq data presented in the figure consists of combined data from HEL47.2 and HEL24.3 lines.

Supplementary Figure 5 Changes in the expression of BMP, Notch and VEGF pathway genes. **(A)** Clusterwise expression of BMP-, Notch-, and VEGF-pathway ligands included in TOP 20 hiPS-CM sender gene list and other BMP- and Notch-ligands that had higher expression in co-culture hiPS-ECs. hiPS-CM and co-culture hiPS-EC clusters are highlighted. Clusterwise expression of **(B)** BMP-, Notch-, and VEGF-pathway receptors and **(C)** BMP- and Notch-pathway targets in monoculture and co-culture hiPS-ECs. All scRNAseq data presented in the figure consists of combined data from HEL47.2 and HEL24.3 lines.

Supplementary Figure 6 Ligand-target gene analysis. **(A)** Expression of top ligands in sender cells (hiPS-EC). **(B)** Ligand–target matrix denoting the regulatory potential between sender (hiPS-EC) ligands and predicted receiver (hiPS-CM) target genes. **(C)** Ligand-receptor interactions between ligands from hiPS-ECs and receptors expressed in hiPS-CMs. All data presented in the figure consists of combined data from HEL47.2 and HEL24.3 lines.

Supplementary Figure 7 Clusterwise expression of ErbB-pathway ligands and receptors. hiPS-CM and co-culture hiPS-EC clusters are highlighted. All scRNAseq data presented in the figure consists of combined data from HEL47.2 and HEL24.3 lines.

Supplementary Figure 8 Clusterwise expression of **(A)** BMP-, **(B)** Notch-, and **(C)** ErbB-pathway genes presented separately in the HEL47.2 and HEL24.3 lines.

Supplementary Figure 9 Results from the independent replication including six pooled hiPS-EC/hiPS-CM co-culture samples. **(A)** The UMAP of the pooled cells. **(B)** Expression of cardiac contractility genes and cardiac secreted protein NPPA coding gene in the EC and CM clusters. Co-culture hiPS-EC expression of **(C)** genes related to heart development and endocardial genes (the same genes as presented in Supplementary Figures 4A,B), and **(D)** BMP-, Notch- and ErbB-pathway genes (the same genes as presented in Supplementary Figures 8A–C).
