# Supplementary material for: HiPS-Endothelial Cells Acquire Cardiac Endothelial Phenotype in Co-culture With hiPS-Cardiomyocytes
Source: Front Cell Dev Biol. 2021 Aug 6;9:715093. doi: 10.3389/fcell.2021.715093 (PMC8378235; doi:10.3389/fcell.2021.715093)
Supplement: Supplementary Figure 1 — Expression of cardiomyocyte markers during differentiation. qPCR analysis shows that CM marker gene expression is increased during differentiation. Two differentiation experiments [HEL47.2 (1.) and HEL47.2 (2.)] with two replicates each are shown. Expression levels have been normalized by comparing to day 1. [file Data_Sheet_1.pdf]

Figure S1

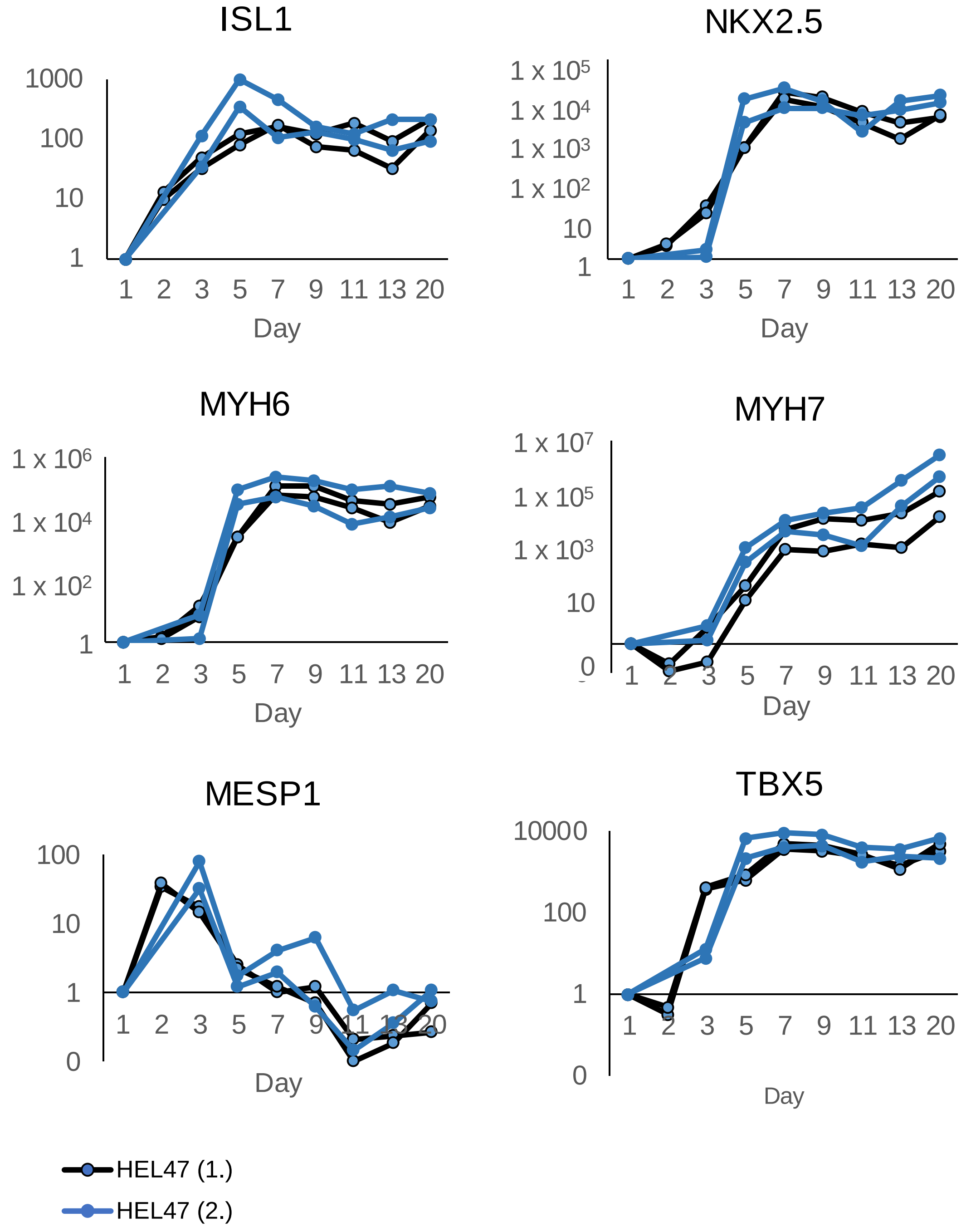

Figure S1: Expression of cardiomyocyte markers during differentiation. qPCR analysis shows that CM marker gene expression is increased during differentiation. Two differentiation experiments (HEL47.2 (1.) and HEL47.2 (2.)) with two replicates each are shown. Expression levels have been normalized by comparing to day 1.

Figure S2

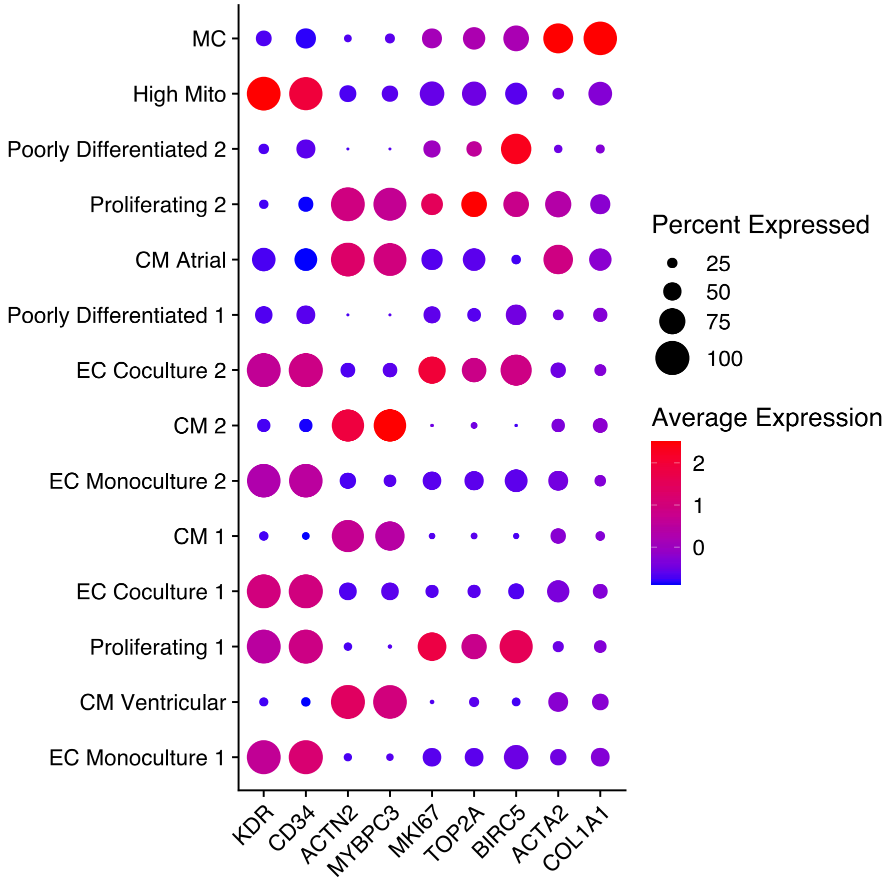

Figure S2. EC, CM, cell cycle and mesenchymal cell gene expression in the defined clusters. All scRNAseq data presented in the figure consists combined data from HEL47.2 and HEL24.3 lines.

Figure S3

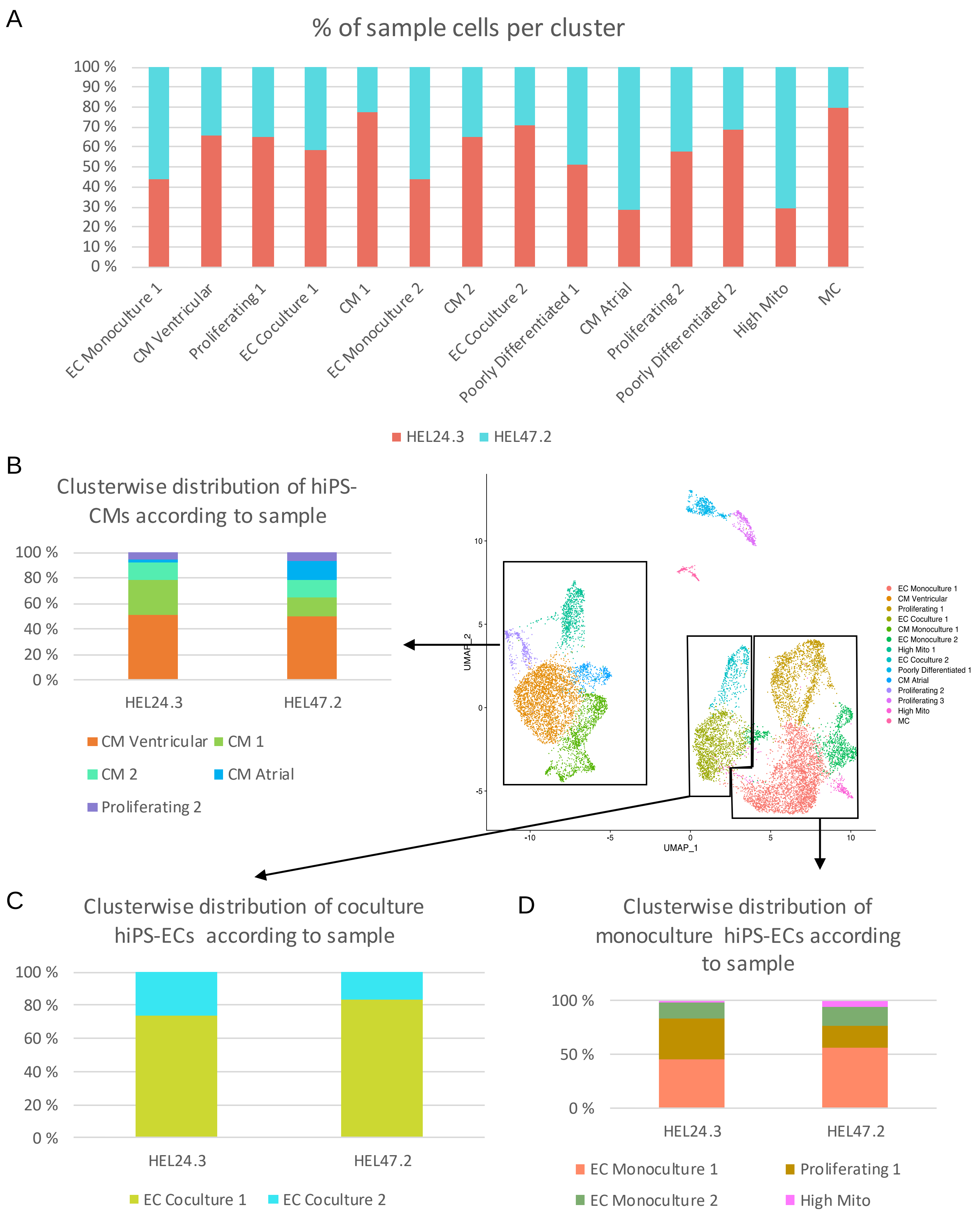

Figure S3. Comparison of hiPS transcriptomics between HEL47.2 and HEL24.3 lines. **A)** Samplewise distribution of cells per cluster. Distribution of cells per cell type cluster according to sample in **B)** hiPS-CM, **C)** hiPS-EC in coculture, **D)** hiPS-EC in monoculture.

Figure S4

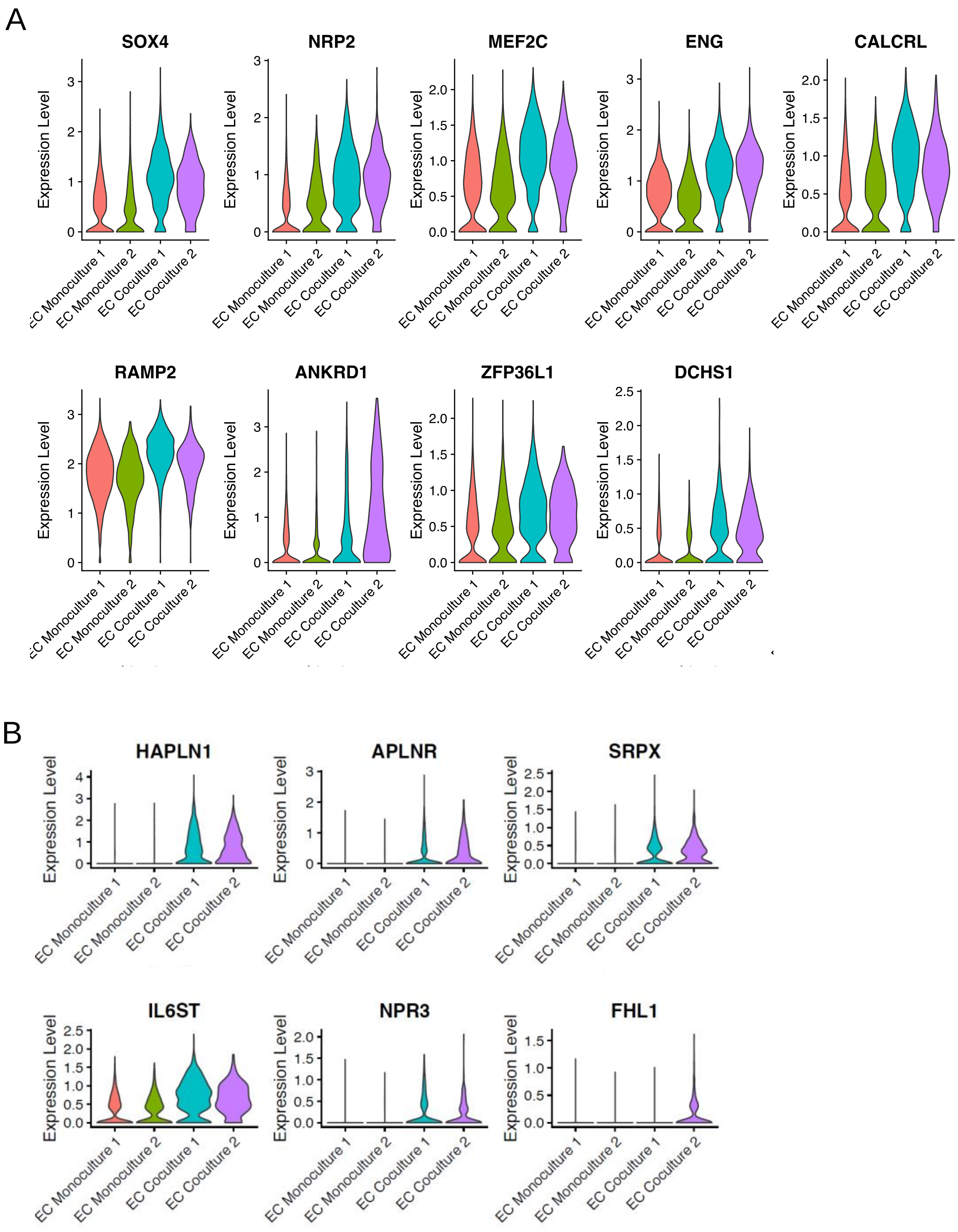

Figure S4. Coculture vs. monocultre hiPS-EC expression of **A)** genes related to heart development, **B)** cardiac EC genes. All scRNAseq data presented in the figure consists of combined data from HEL47.2 and HEL24.3 lines.

Figure S5

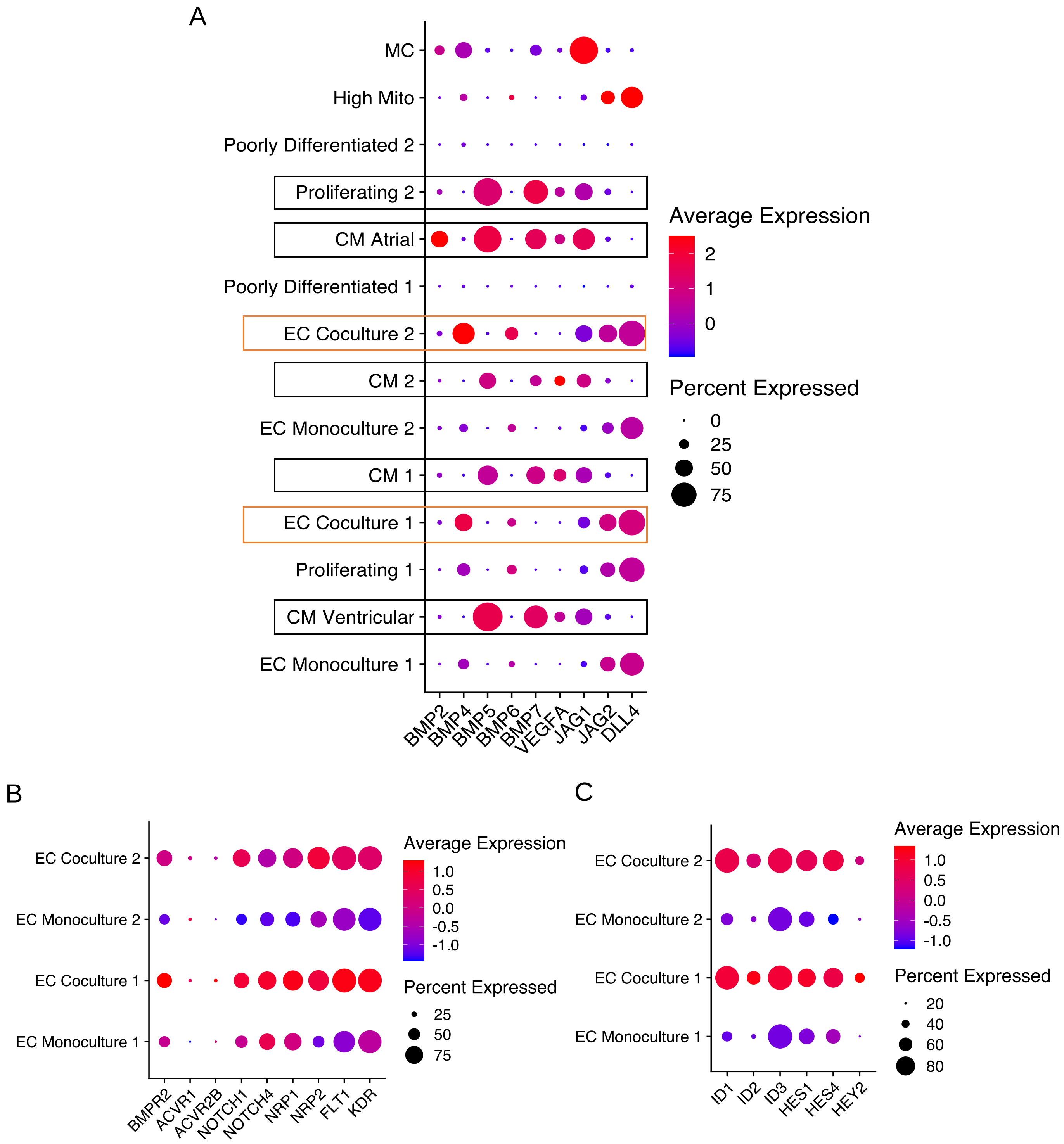

Figure S5. Changes in the expression of BMP, Notch and VEGF pathway genes. **A)** Clusterwise expression of BMP-, Notch-, and VEGF -pathway ligands included in TOP 20 hiPS-CM sender gene list and other BMP- and Notch-ligands that had higher expression in coculture hIPS-ECs. hiPS-CM and co-culture hiPS-EC clusters are highlighted. Clusterwise expression of **B)** BMP-, Notch-, and VEGF -pathway receptors and **C)** BMP- and Notch -pathway targets in monoculture and coculture hiPS-ECs. All scRNAseq data presented in the figure consists of combined data from HEL47.2 and HEL24.3 lines.

Figure S6

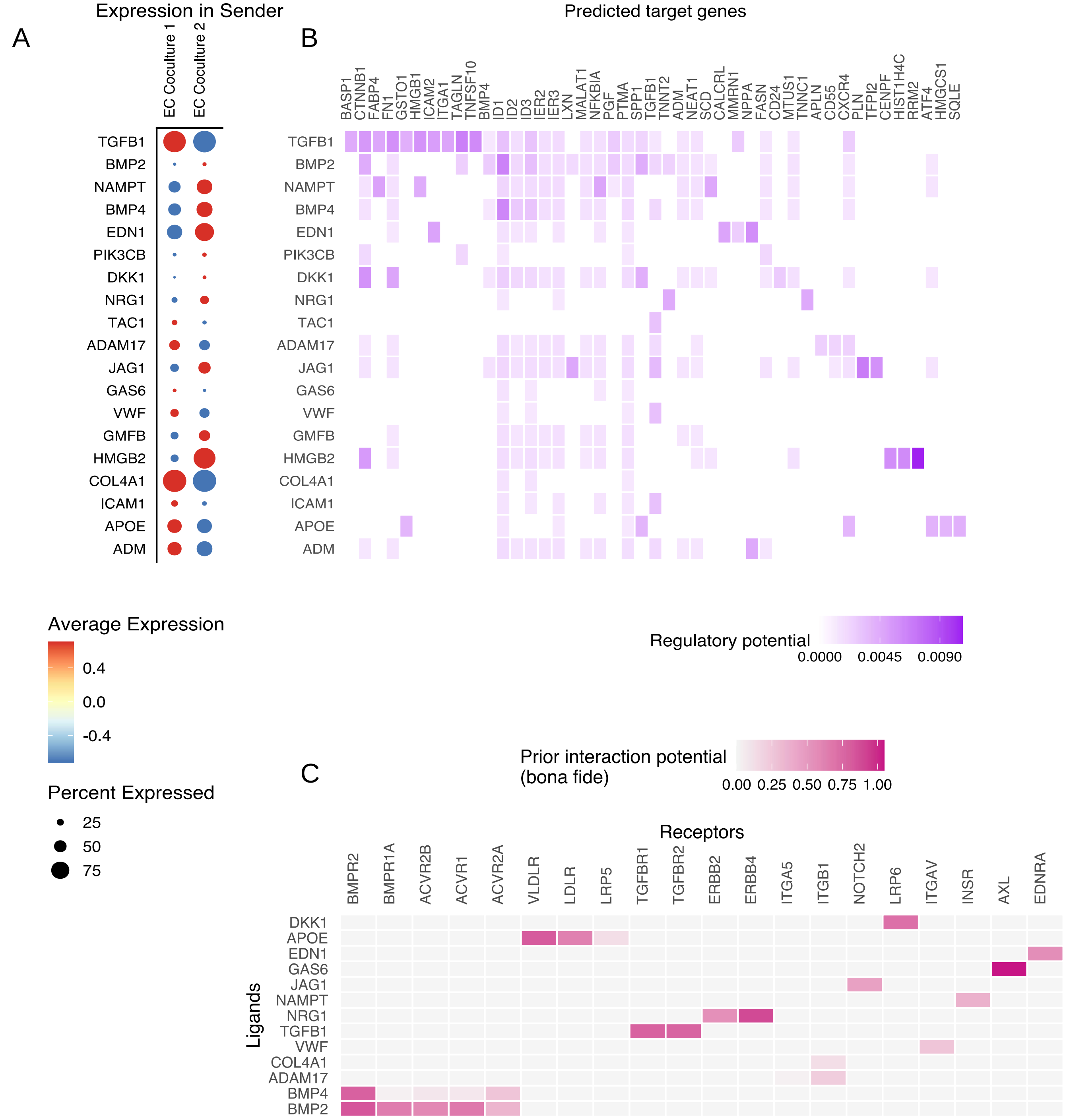

Figure S6. Ligand-target gene analysis. **A)** Expression of top ligands in sender cells (hiPS-EC). **B)** Ligand–target matrix denoting the regulatory potential between sender (hiPS-EC) ligands and predicted receiver (hiPS-CM) target genes. **C)** Ligand-receptor interactions between ligands from hiPS-ECs and receptors expressed in hiPS-CMs. All data presented in the figure consists of combined data from HEL47.2 and HEL24.3 lines.

Figure S7

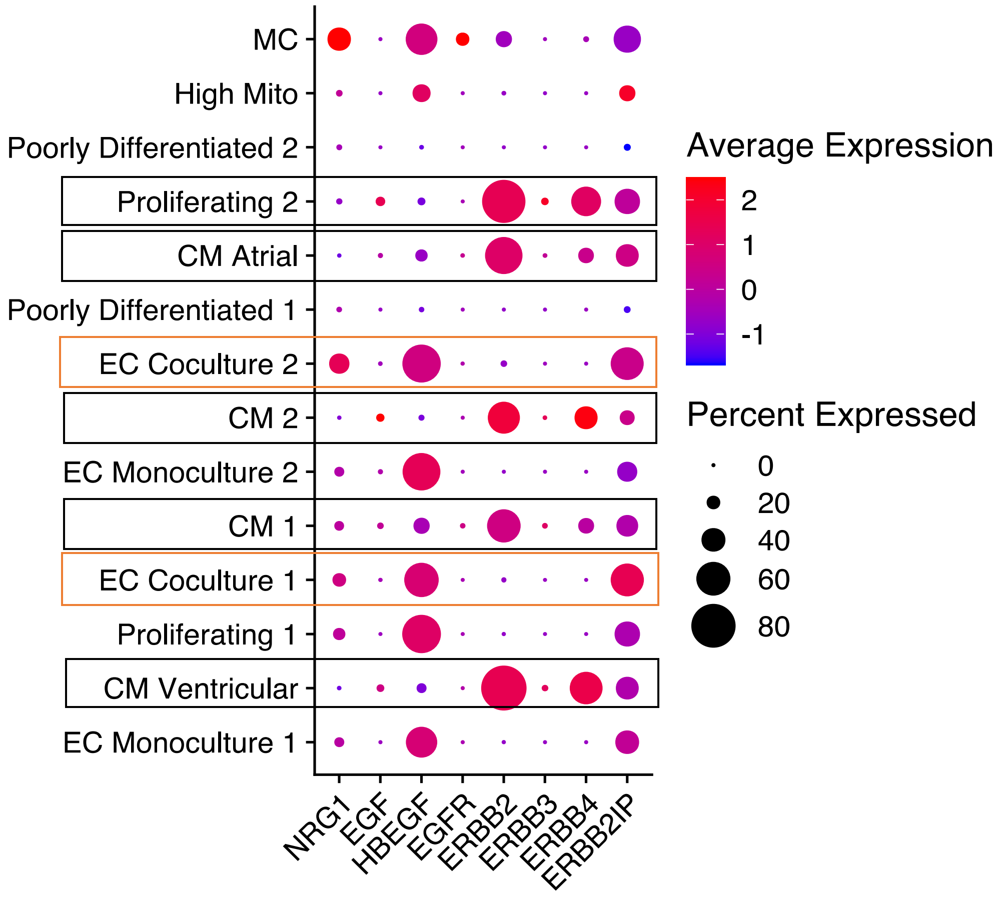

Figure S7. Clusterwise expression of ErbB-pathway ligands and receptors. hiPS-CM and co-culture hiPS-EC clusters are highlighted. All scRNAseq data presented in the figure consists of combined data from HEL47.2 and HEL24.3 lines.

Figure S8

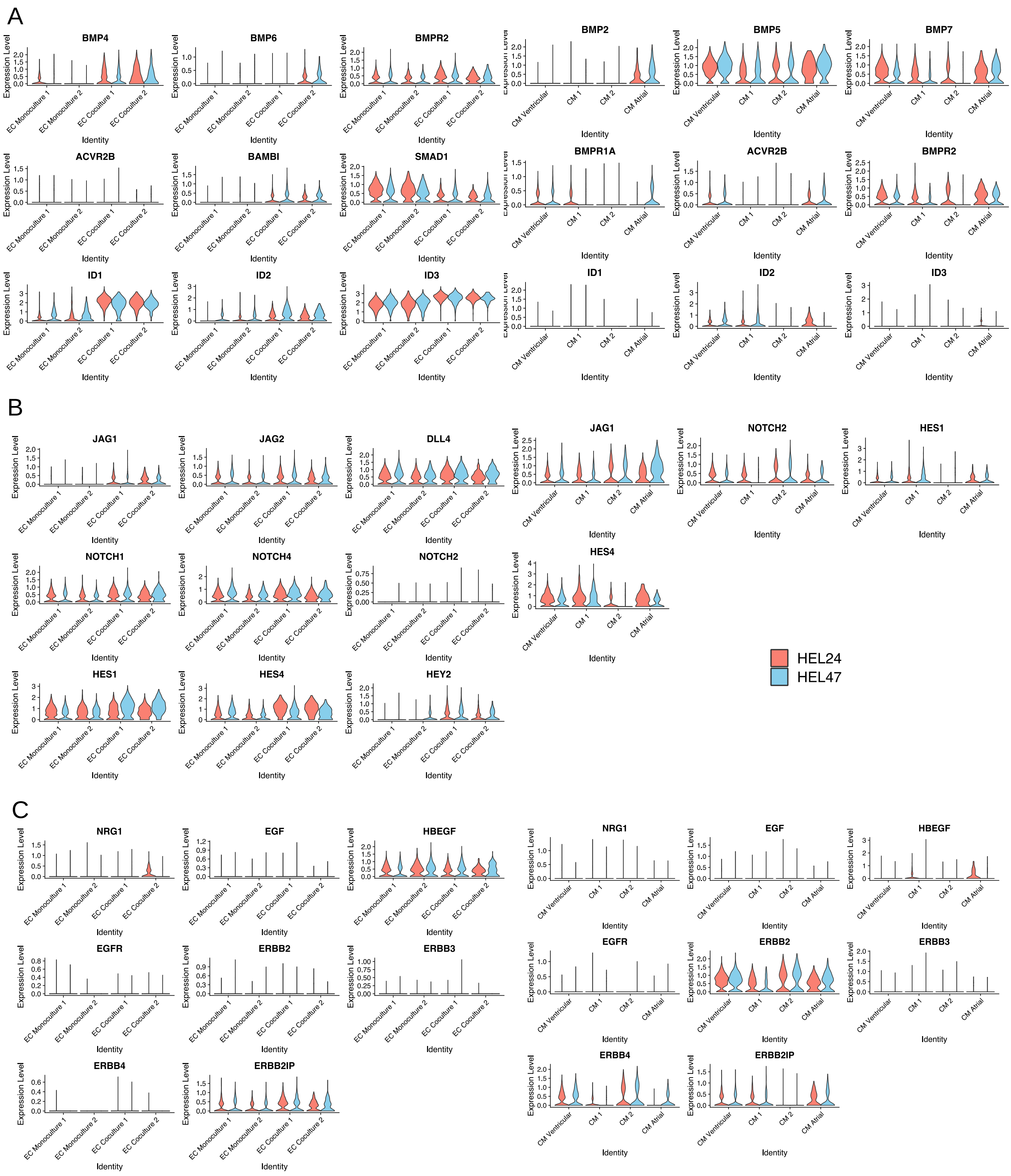

Figure S8. Clusterwise expression of **A)** BMP-, **B)** Notch-, and **C)** ErbB-pathway genes presented separately in the HEL47.2 and HEL24.3 lines.

Figure S9

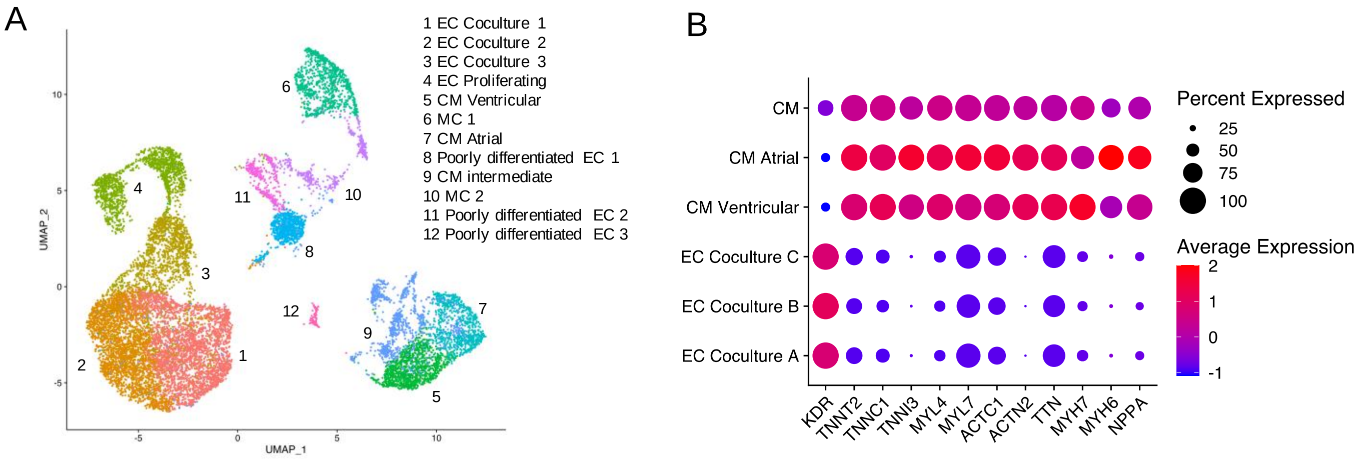

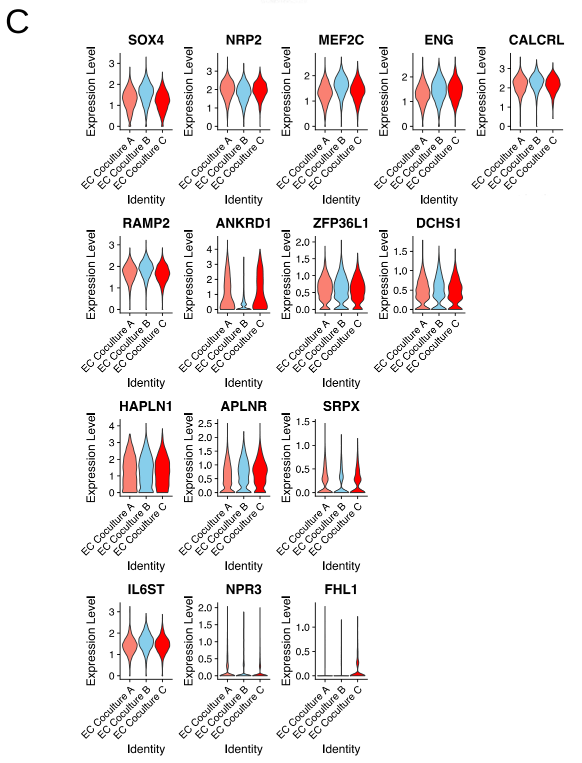

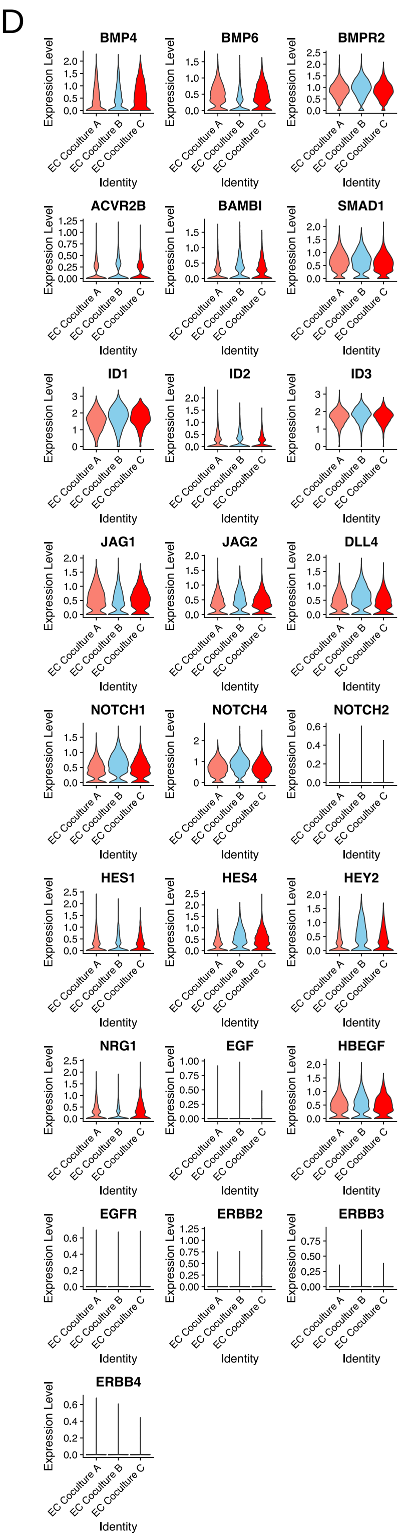

Figure S9. Results from the independent replication including six pooled hiPS-EC/hiPS-CM coculture samples. **A)** The UMAP of the pooled cells. **B)** Expression of cardiac contractility genes and cardiac secreted protein NPPA coding gene in the EC and CM clusters. Coculture hiPS-EC expression of **C)** genes related to heart development and endocardial genes (the same genes as presented in Figure S4A-B), and **D)** BMP-, Notch- and ErbB-pathway genes (the same genes as presented in Figure S8A-C).
